# Fine Resolution Analysis of Microbial Communities Provides Insights Into the Variability of Cocoa Bean Fermentation

**DOI:** 10.3389/fmicb.2020.00650

**Published:** 2020-04-15

**Authors:** Mauricio Edilberto Pacheco-Montealegre, Lizeth Lorena Dávila-Mora, Lina Marcela Botero-Rute, Alejandro Reyes, Alejandro Caro-Quintero

**Affiliations:** ^1^Corporación Colombiana de Investigación Agropecuaria - AGROSAVIA sede Tibaitatá, Mosquera, Colombia; ^2^Grupo de Biología Computacional y Ecología Microbiana BCEM - Max Planck Tandem Group in Computational Biology, Universidad de los Andes, Bogota, Colombia

**Keywords:** amplicon sequencing, cocoa bean fermentation, fermentation heterogeneity, microbial dynamics, oligotypes

## Abstract

Cocoa bean fermentation is an important microbial process, where most metabolites that affect chocolate quality and aroma are generated. Production of reproducible high-quality beans is a major challenge because most fermentations occur in open containers with a lack of variable control. Here we present a study that aims to identify the effect of farm protocols, climate, and bean mass exposure, in the dynamics and composition of microbial communities. Using high-throughput sequencing of molecular markers for bacteria and yeasts, complemented with culture-based methods, we evaluated the microbial diversity and dynamics associated to spontaneous cocoa fermentation in two distinct agro-ecological zones in Colombia. The bacterial communities were classified at two levels of evolutionary relationship, at a coarse resolution (OTU-level) and at a finer resolution (oligotype-level). A total of six bacterial OTUs were present in both farms, following a microbial succession that starts with the *Enterobacteraceae* family (one OTU), transitioning to the *Lactobacillaceae* family (three OTUs), and finishing with *Acetobacteraceae* family (two OTUs). When undesirable practices were done, OTUs were observed at unexpected moments during the fermentation. At a finer taxonomic resolution, 48 oligotypes were identified, with 46 present in both farms. These oligotypes have different patterns of prevalence. In the case of *Lactobacillaceae* a high evenness was observed among oligotypes. In contrast, for *Enterobacteraceae* and *Acetobacteraceae* a high dominance of one or two oligotypes was observed, these oligotypes were the same for both farms, despite geographic location and season of sampling. When the overall fermentations were compared using correlations matrices of oligotypes abundance, they show a clear clustering by farm, suggesting that farm protocols generate a unique fingerprint in the dynamics and interactions of the microbial communities. The comparison between the upper and middle layers of the bean mass showed that environmental exposure affects the paces at which ecological successions occur, and therefore, is an important source of cocoa quality heterogeneity. In conclusion, the results presented here showed that the dynamics of microbial fermentation can be used to identify the sources of variability and evidence the need for better fermentation technologies that favor the production of reproducible high-quality cocoa beans.

## Introduction

Cocoa bean fermentation is a spontaneous process where microorganisms transform physically and biochemically all bean structures (husk, mucilage, cotyledon, and embryo). The fermentation process lasts 3–7 days depending on variables such as the genetic origin of the seed, the agro-ecological conditions and the protocol used. These microbial communities are composed mainly by yeasts, *Enterobacteriaceae* related bacteria (ENT group), Lactic Acid Bacteria (LAB group), and Acid Acetic Bacteria (AAB group) (Camu et al., 2007; Okiyama et al., 2017). During the first hours, yeasts degrade the cocoa pulp converting carbohydrates into ethanol and lowering pulp viscosity, on the other hand, the ENT has a pectinolytic activity. Subsequently, LAB degrades the carbohydrate-rich pulp producing mainly lactic acid, finally, AAB oxidizes ethanol into acetic acid (Kongor et al., 2016; Ozturk and Young, 2017). In this last step, the production of acetic acid lowers pH and increases bean mass temperature (40–50°C), these conditions affect the seed and cause its death, promoting the subsequent release of the flavor precursors associated with chocolate quality (Schwan et al., 2014; De Vuyst and Weckx, 2016; Illeghems et al., 2016). Microorganisms are also responsible for the production of volatile compounds related to aromatic flavor (e.g., alcohols, organic acids, esters, and aldehydes) and the removal of compounds associated with bitterness and astringency (e.g., tannins, polyphenols) (Hamdouche et al., 2015; Kongor et al., 2016; Okiyama et al., 2017). Despite the understanding of the fermentation succession and function, the production of reproducible high-quality beans still a major challenge for farmers, because most fermentations occur in open containers with a lack of variability control and without local standardization of fermentation protocols.

The dynamics of microbial communities are strongly related to the transformation of cocoa seeds and therefore, the dynamics of the microbial composition can be used as an indicator to identify the main sources of variability during the transformation of cocoa beans. To follow in more detail *in vivo* fermentation processes, culture-independent techniques have been used to follow the changes of the microbial communities and identify the main bacterial species involved in the transformation of pulp and seed. Techniques such as PCR-DGGE (Denaturing gradient gel electrophoresis), gene clone libraries, PCR-RFLPs (Restriction Fragment Length Polymorphism) (Garcia-Armisen et al., 2010), and lately 16S rRNA gene and ITS amplicon sequencing (Mota-Gutierrez et al., 2018; Papalexandratou et al., 2019; Serra et al., 2019) have been used to identify the species and how they change throughout the fermentation process.

Recently, metagenomic shotgun sequencing has been used to evaluate the composition and metabolic potential of the community at one-time point of the fermentation (Blaxter et al., 2005), by extracting the microbial community DNA from different samples (Agyirifo et al., 2019), revealing a wider diversity of bacteria and yeasts, and elucidating the contributions of the distinct functional groups in the biochemical transformation of cocoa bean mass. These results suggest that culture-independent techniques are valuable tools to characterize the changes in microbiota composition and the transformation of the cocoa seeds.

In recent years, it has become evident that closely related organisms can have different adaptations and functional roles in the ecosystems. In cocoa fermentation, the identification of this fine diversity might be important to understand, modulate and standardize the fermentation process. For instance, it was identified that distinct LAB strains have differences in the use of external electron acceptors and their activity during cocoa fermentation (Adler et al., 2013). Identification of this fine level diversity can be accomplished by coupling high-throughput sequencing of molecular markers and variant detection. This coupled methodology allows a fine-scale monitoring of compositional changes of the community tackling the limitations of previously used methods such as gel-based profiling and is more cost-effective compared to shotgun metagenomics. In the case of high-throughput sequencing of the 16S rRNA gene phylogenetic marker, species are defined as Operational Taxonomic Units (OTUs) (Blaxter et al., 2005), clustered at 97% sequence identity (Schloss et al., 2009; Caporaso et al., 2010). Identification of variants within each OTU is a more challenging task that cannot be achieved by identity thresholds but by the use of newer computational analysis methods such as oligotyping (Eren et al., 2013). This technique has been successfully implemented in a large variety of ecological studies such as the human oral microbiota (Eren et al., 2014), plant-associated microbiota (Maignien et al., 2014), the study of specific strains associated to infant food allergy (Canani et al., 2016), and Kombucha tea fermentation (De Filippis et al., 2018), among others. These approaches have demonstrated its utility for the identification of ecologically significant subgroups. Recently, oligotyping analysis has been used to study the microbial communities of fermented beverages (Wu et al., 2015), showing that it is possible to monitor closely related microorganisms with different patterns of abundance and ecology.

Here we present a study that explores the effect of farm protocols, climate (dry and wet seasons), and bean mass exposure in the composition of the microbial communities involved in cocoa fermentation in Colombia. To do this, we used high-throughput sequencing of phylogenetic markers to study the microbial communities associated to the cocoa bean fermentation, selecting two farms located in distinct agro-ecological zones (AEZs) from Colombia (Federación Nacional de Cacaoteros, 2013; Evaluaciones Agropecuarias Municipales, 2016), *Montaña Santandereana* (MS) and *Zona Marginal Baja Cafetera* (BC). In these farms, we evaluated the fine level diversity of the microbial communities throughout the fermentation process and establish their dynamics and its relationship with geographic location, climate, and fermentation protocols. Such understanding has important implications for the improvement of fermentation technologies, the optimization of protocols and might be relevant to monitor and selection of potential starter cultures (Kongor et al., 2016; Magalhães da Veiga Moreira et al., 2017; Ozturk and Young, 2017).

## Materials and Methods

### Sample Collection

One farm was selected from each AEZ, *Montaña Santandereana* (MS) and *Zona Marginal Baja Cafetera* (BC) to conduct the study. The criteria for selection was based on several factors including, the certification of Good Agricultural Practices (GAP), the existence of clear protocols of fermentation, and validation of fermentation through the monitoring of the structure and color of fermented beans using the cut-test (Okiyama et al., 2017) and determination of the fermentation index (see Table S1). The sampling of the cocoa beans mass was done in both farms at two different times during the wet and dry season in 2016. Cocoa pods were harvested using brier hook tool or pruning shears, beans were removed by hand (Pulp-preconditioning phase) and placed in a fermentation wooden box and loaded with cocoa beans. To monitor the taxonomic composition of the microbial communities, 100 g of cocoa beans were collected at each of two different depths (upper and middle section) of the fermenter box, using sterile gloves, during the whole fermentation process (6–7 days). Samples were collected every 12 h and immediately stored at −20°C. A total of 94 cocoa bean samples were collected (see Figure S1).

### DNA Extraction From Bean Mass

The microbial cells were recovered from the bean samples according to the protocol of Camu et al. (2007), with some modifications. Twenty grams of frozen beans pulp samples were homogenized with 0.85 g/100 ml of NaCl in 250 ml Erlenmeyer flasks in a rotatory shaker (Thermo Scientific MaxQ™ 4000 Invitrogen, Burlington, Ont., Canada); 150 rpm for 30 min. The generated fluid was decanted and filtered through sterile gauze. The free-pulp solution was centrifuged at 3,220 × g at 12°C for 20 min (Hermle, Z32HK, Labortechnik GmbH, Wehingen, Germany) to remove large particles. The biomass was resuspended in 10 ml of sterile saline solution. The DNA was extracted using the UltraClean Microbial DNA isolation kit (MoBio, Carlsbad, CA, USA) and the concentration and purity of DNA was quantified using a NanoDrop™ 1000 Spectrophotometer (Thermo Fisher Scientific, DE, USA). The final community DNA samples were stored at −20°C until further use.

### Amplicon Library Preparation and Sequencing

The analysis of the microbial communities associated with the cocoa bean mass was done by sequencing of phylogenetic markers. For bacteria, the hypervariable V4 region of the 16S rRNA gene was amplified, while for yeasts the region ITS2 from the 5.8S—LSU operon was used. Bacterial and yeast DNA libraries were prepared according to Faith et al. (2013). In brief, the sequencing library preparation was carried out in a two-step PCR procedure. In the first PCR, the modified primers 515F-806R (Caporaso et al., 2011) and ITS4_KY03—ITS3_KY02 (Toju et al., 2012) were used for bacteria and yeasts, respectively. Primer modification includes a linker region in the 5′ end of the primer, as shown in Table S2. During the second PCR, barcodes are added to the amplicons as well as the Illumina i5 and i7 regions (Caro-Quintero and Howard, 2015).

The first PCR, for both genes, was performed in triplicate and carried out in 25 μl reaction volumes. For ITS, each reaction contained 0.1 μl Platinum™ *Taq* DNA Polymerase High Fidelity (Invitrogen Carlsbad, CA, USA), 0.75 μl MgS0_4_ 50 mM, 2.5 μl Buffer-Mg 10X, 0.5 μl dNTPs 10 mM, 0.5 μl (10 μM) of each forward and reverse primers with adaptor sequences, 2 μl of DNA and 18.15 μl of Ultrapure Distilled Water (Invitrogen Carlsbad, CA, USA).

In the case of the 16S rRNA gene, the first PCR (T100™ Thermal Cycler, BioRad, CA, USA) consists of initial denaturation at 94°C for 120 s, followed by 35 cycles of denaturation at 94°C for 45 s, annealing at 50°C for 60 s and extension at 72°C for 90 s, and a final extension of 72°C for 10 min. In the case of the ITS, the first PCR consists of an initial denaturation at 95°C for 120 s, followed by 35 cycles of denaturation at 95°C for 30 s, annealing at 55°C for 30 s and extension at 72°C for 60 s, and a final extension of 72°C for 5 min. All PCR products were purified following the protocol provided by Agentcourt^®^ Ampure^®^ XP (Beckman Coulter, Brea, CA, USA)

The second PCR used to add the barcodes to the 16S rRNA amplicons was carried out by adding 5 μl of purified product, 1 μl (10 μM) of each of the forward and reverse indexed primers, 0.1 μl Platinum™ *Taq* DNA Polymerase High Fidelity (Invitrogen Carlsbad, CA, USA), 0.75 μl MgS0_4_ 50 mM, 2.5 μl Buffer 10X, 0.5 μl dNTPs 10 mM (100 mM dNTP set, Invitrogen, Carlsbad, CA, USA). In the case of ITS, the PCR reaction was prepared as follow, 5 μl of purified product, 1 μl (10 μM) of each of the forward and reverse indexed primers, 0.1 μl Platinum™ *Taq* DNA Polymerase High Fidelity (Invitrogen Carlsbad, CA, USA), 1.5 μl MgS0_4_ 50 mM, 2.5 μl Buffer 10X, 0.5 μl dNTPs 10 mM, 13.4 μl of Ultrapure Distilled Water (Invitrogen Carlsbad, CA, USA). PCR cycle for both genes was performed using the same conditions as described above for the first PCR of the 16S rRNA gene but allowed to proceed for only 12 cycles. The PCR products from both amplifications were analyzed on agarose gels (1.5%) after staining with SYBR Safe (Invitrogen Carlsbad, CA, USA). Products of the second PCR were purified with AMPure XP beads (Beckman Coulter, Brea, CA), and DNA concentrations were quantified on a NanoDrop™ 1000 Spectrophotometer (Thermo Fisher Scientific, DE, USA). Samples were pooled to equimolar concentrations and pair-end sequenced (250 nt PE reads) on the Illumina MiSeq at the Microbial genomics laboratory of the Molecular Genetics and Antimicrobial Resistance Unit at Universidad El Bosque, Bogotá, Colombia.

### Isolation of Bacteria Involved in Fermentation

Cocoa bean samples for bacteria and yeast isolation were collected aseptically from the center of the fermentation bean mass. Around 10 g were sampled every 24 h during the fermentation process. Each sample was mixed with 90 mL of sterile saline solution (0.85% of NaCl), from this solution, 10^−1^, 10^−3^, and 10^−5^ serial dilutions were prepared. From each serial dilution, 0.1 mL was inoculated on solid culture media. The MRS agar (Merck KGaA, Darmstadt, Germany) was used for Lactic Acid Bacteria (LAB), incubated at 39°C for 16 h. In the case of AAB, the serial dilution was plated on GYEC pre-selective media (Pereira et al., 2013b), incubated at 30°C for 48 h.

For the LAB group, representative colonies with differential morphotypes were selected from the agar plates with the highest dilution. Each colony was transferred to MRS broth (Merck KGaA, Darmstadt, Germany) and streaked in the solid media to verify purity and the presence of clearly isolated morphotypes. For the AAB group, colonies that grew on the GYEC agar (10^−5^ dilution plate) were scrapped and resuspended in acetic acid bacteria pre-enrichment broths (Pereira et al., 2013b), broths were incubated at 30°C for 5 days under continuous shacking (180 rpm). After incubation, pre-enrichment broths were streaked on GYE 1 agar and GYE 2 agar and incubated at 30°C for 3 days (Table S3), the presence of solubilization rings around colonies indicated the presence of AAB. In order to assure that colonies were AAB, these were transferred again to pre-enrichment broths, and after incubation, the isolation of colonies was performed by plating for 3 days at 30°C in GYE 1, GYE 2, *A. aceti* agar (DSMZ, 2007c), *A. tropicalis* agar (DSMZ, 2007a), *A. ghanensis* agar (DSMZ, 2007b), and Gluconacetobacter agar (Atlas, 2010). For the obtained colonies a basic characterization for morphology under the contrast microscope, gram staining, and catalase test were performed.

### Bacterial Colonies DNA Extraction and Molecular Identification

Each isolated colony was transferred to 2 ml microtubes to perform DNA extraction using the ZR-96 Fungi/Bacterial DNA kit from Zymo Research (Irvine, CA, USA), following the manufacturer's instructions. The DNA concentration and purity were determined with Nanodrop equipment and electrophoresis in a 1 g/100 ml agarose gel.

PCR amplification for the 16S rRNA gene was performed using the following primers 27F 5′ AGAGTTTGATCMTGGCTCAG-3′, 1492R 5′- AGAGTTTGATCMTGGCTCAG-3′. The PCR was performed in a volume of 25 μl. Each reaction was composed of 2.5 μl of buffer 10x, 1 μl 50 mM MgSO_4_, 0.5 μl of 10 mM DNTP mix (2.5 mM each, Invitrogen, Carlsbad, CA), 0.5 μl of each primer, 0.1 μl of Platinum™ *Taq* DNA Polymerase High Fidelity (Invitrogen, Carlsbad, CA), and 2 μl of template DNA. The PCR program consisted of 2 min at 94°C followed by 10 cycles of 15 s at 94°C, 30 s at 46°C and 1 min at 72°C, followed by 20 cycles of 15 s at 94°C, 30 s at 50°C, 2 min at 72°C, with a final 7 min extension at 72°C. The amplified products were visualized using electrophoresis 2 μl of amplified PCR product in a 1.5 g/100 ml agarose gel. The PCR products were sequenced by Macrogen (Korea).

### Bioinformatics Analysis

#### Pre-processing of Reads

MiSeq sequencing reads were uploaded to the HPC system at Universidad de los Andes. Quality inspection was performed using FastQC v. 0.11.2. The trimming was done with Trimmomatic v. 0.36, removing primers and nucleotides with a low-quality score. Demultiplexing and pair-end assembly were performed using QIIME v 1.9.1 scripts (join pair-end reads and split libraries) with high stringency in specific parameters (Table S4).

#### Operational Taxonomic Unit (OTU) Determination

We used QIIME version 1.9.1 to perform *de novo* OTU clustering with UCLUST method (Caporaso et al., 2010) for both molecular markers. Subsequently, we perform taxonomic assignment against the Greengenes database (version 13.8, http://greengenes.lbl.gov), and created a biom table where we filter chloroplast and OTUs with relative abundance below 1%, for 16S rRNA gene reads (parameters are included in Table S4). For the ITS reads we used NCBI-BlastN against the nucleotide non-redundant (nt) database to perform the taxonomic assignment.

#### Oligotyping Analysis

Oligotyping v. 2.1 (http://merenlab.org/software/oligotyping/) was used for the generation of fine taxonomic resolution groups, for this, all reads of an OTU were selected and aligned using PyNAST, as implemented in QIIME. Subsequently, we stripped common gaps from each alignment following the suggested pipeline (http://merenlab.org/2012/05/11/oligotyping-pipeline-explained/), and the Shannon entropy was calculated for each base position in the alignment (Eren et al., 2013). Finally, to resolve all oligotypes in a bacterial taxon, we used all available highly variable base positions for each OTU. For this purpose, we selected specific Shannon-entropy cut-offs (Table S5) for each OTU and all positions in the alignment that presented equal or better value were selected as components (c parameter) for the oligotype script in the pipeline.

An oligotype was considered if it was present in more than 10% of all reads (a = 10) and if the minimum number of samples containing the oligotype was more than 10 (s = 10). The counts of each oligotype were normalized relative to the total number of 16S rRNA gene reads per sample. We used the package *pheatmap* in R for visualization purposes.

To evaluate the abundance relationship between all oligotypes from the study, we used the Spearman's correlation coefficients (rs) with *Hmisc* and using *corrplot* for visualization in R.

### Phylogeny Between Oligotypes and Isolates

The taxonomic relationship between the oligotypes and isolates was inferred aligning the V3–V4 region of the 16S rRNA gene. The alignment was done with MUSCLE (Edgar, 2004) using the Neighbor-Joining method. The tree reconstruction was conducted with SeaView (Gouy et al., 2010). The bootstrap consensus tree was inferred from 1,000 replicates and is taken as a representation of the taxonomic relationship of the taxa analyzed. All positions containing gaps and missing data were eliminated. A total of 299 positions were obtained in the final dataset. The final alignment is provided as Supplementary Information.

## Results

### Fermentation Protocols Varied Between Farms

Cocoa plantations in Colombia are distributed throughout the country and each productive region is delimited in agro-ecological zones (AEZ) based on climatic conditions, topography, and soil composition. We choose two of the most relevant AEZ, (i) *Montaña Santandereana* (MS), the region with the largest production rates of cocoa in the country, and (ii) *Zona Marginal Cafetera Baja* (BC), one of the most productive regions with low temperature and humidity rates (Federación Nacional de Cacaoteros, 2013). For each AEZ 12 farms were visited, and only one farm was selected for sampling. The criteria for selection were based on several factors as described in Table S1, where the main goal was to select farms with the best agricultural practices and with a well-established infrastructure and knowledge on cocoa fermentation.

Even though the two selected farms were selected as the ones with the best practices, a detailed analysis of the fermentation protocols showed that the fermentation protocols between farms were heterogeneous, and even changed within farms during the sampling season. The most notorious differences were the length of the process and the timing for the initiation of the bean mixture. In MS, the length of the fermentation process was in general shorter 108 h in March (dry season), and 132 h in June (wet season), while in BC farm fermentation lasted 132 h in May (dry season) and 144 h in June (wet season) (Figure S1). Mixing of cocoa beans also started at different times, 48 h in May (dry season) and 24 h in June (wet season), while for BC while it started at 24 h for both climate regimes in MS.

### Sampling and Amplicon Sequencing

Cocoa bean samples were collected throughout one complete fermentation process during two different climate regimes (wet and dry season). Sampling was performed every 12 h and at two depths on each farm. Overall, 94 samples were collected for the microbiota analysis, 44 samples from MS and 50 from BC farm (Figure S1).

Amplicon libraries for the 16S rRNA gene and fungal ITS region were prepared and sequenced for each sample (Table 1). A total of 2,742,198 reads were obtained for 16S rRNA gene libraries. After quality control, 1,426,240 reads were kept for further analysis. In the case of ITS sequencing, 443,340 reads were obtained initially, and 60,339 reads were kept after quality control (Table S4).

**Table 1:** Summary of the sequencing effort during the microbial monitoring study of the cocoa bean fermentation.

		**Zona marginal baja cafetera**				**Montaña santandereana**			
		**Dry season May**		**Wet season June**		**Dry season March**		**Wet season June**	
		**Upper zone**	**Middle zone**	**Upper zone**	**Middle zone**	**Upper zone**	**Middle zone**	**Upper zone**	**Middle zone**
Bacteria	Number of raw sequences	330,892	263,452	297,372	338,781	295,059	274,659	480,765	461,218
	Number of clean sequences	242,225	185,386	224,372	249,150	214,897	213,455	345,905	314,520
	% of reads assigned to cocoa tree^*^	3.21	5.06	4.14	4.18	7.92	0.99	5.02	7.11
	Number of Oligotypes	44	45	47	44	42	48	48	48
Yeast	Number of raw sequences	83,678	64,715	66,344	62,764	56,342	32,586	34,624	42,287
	Number of clean sequences	10,014	10,857	12,564	10,330	9,619	5,985	5,935	5,365

* *The value was calculated from the number of clean reads*.

In order to determine the saturation of the sequencing effort, a rarefaction analysis was done for all individual libraries of 16S rRNA gene and ITS at the OTU level. The result showed that for both markers, the diversity present in the samples was well-covered (Figure S2). A similar result was obtained for the ITS libraries (data not shown), showing that the obtained reads were also enough to saturate the yeasts and mold diversity present in the samples.

### The Fermentation Process Is Governed by a Low Number of Species, Independent of the Site and Time of Year

**Figure 1:**
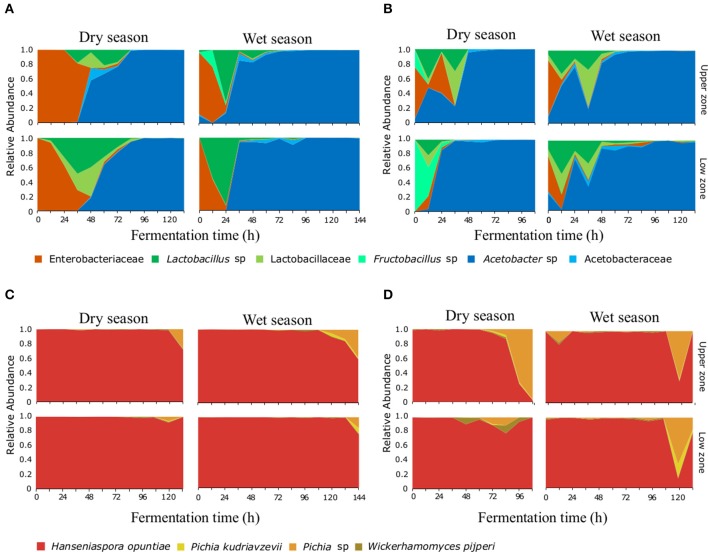
Microbial succession of bacteria and yeasts, during the fermentation process of cocoa beans. A total of six OTUs for bacteria **(A,B)** and four OTUs for yeasts **(C,D)** were detected. Relative abundance for each OTU at the different agro-ecological zones [BC farm **(A,C)** and MS farm **(B,D)**] on each depth and month surveyed are shown during the first 140 h of the fermentation process.

The bacterial taxonomic analysis identified six bacterial OTUs (97% identity) and one *Theobroma cacao* chloroplast OTU (in both farms). Approximately 5% of the processed 16S rRNA gene reads were assigned to chloroplasts and were present mainly during the first 24 h of the fermentation process (Figure S3), these reads were removed from the microbial community analysis, but their abundance was used later to elucidate patterns associated to practices and protocols. During the initial hours of the fermentation, the most abundant bacterial species corresponded to an OTU assigned to the *Enterobacteriaceae* family (Figures 1A,B). The Lactic Acid Bacteria became dominant mainly at the 24–36 h of the fermentation, this group was represented by three distinct OTUs assigned to the genus *Lactobacillus* sp., the family *Lactobacillaceae*, and the genus *Fructobacillus* sp. Finally, the Acetic Acid bacteria, dominated after the 48 h (in both farms), these group was represented by two OTUs assigned to the genus *Acetobacter* sp. and the *Acetobacteraceae* family. Regarding the ITS assignments, four OTUs at the 95% identity-level were detected throughout the fermentation process (Figures 1C,D). *Hanseniaspora opuntiae* represents more than 80% of the yeast's relative abundance at any time during the fermentation process. *Pichia* sp., *Pichia kudriavzevii*, and *Wickerhamomyces pijperi* increased slowly and appeared only in the final stages of the fermentation process in both AEZs.

### Fermentation Protocols Affect the Succession of the Microbial Communities

The bacterial composition during the fermentation process showed differences between farms, especially in the transition (i.e., the time when the LAB group is replaced by AAB in the succession) and dominance of bacterial groups. In the BC farm during the dry season sampling (Figure 1A) the ENT group was present during the first 36 h, being dominant during the initial 12 h, the LAB group was dominant at 48 h and the AAB group was dominant from the 56 h to the end of the process. While in the rainy season sampling, the ENT was present only during the first 24 h. LAB showed one clear peak of abundance at 24 h, while AAB was dominant after the 36 h. In the MS farm during the dry season sampling, the ENT was present during the first 36 h (Figure 1B), showing two peaks of dominance at 12 and 36 h. Finally, the AAB group was dominant after 48 h and until the end of the process. In June, the ENT group was present during the first 12 h, the LAB group was present from 0 to 48 h, and similarly to what happened in March, two peaks of abundance were observed. Finally, the AAB was dominant from the 48 h to the end of the process.

The variation in the transition times of the microbial succession seems to be affected directly by the fermentation protocols used. For instance, the two farms start bean mixing (the incorporation of oxygen) at different time points (e.g., 24 h in MS and 48 h in BC) which affects the transition of the LAB to the AAB group. The effect of the mixing is clearly shown by the relationship between the increase in temperature and the increase in AAB group abundance (Figure S4). Another fermentation practice that affected the dynamics of the microbial communities is the posterior addition of fresh bean mass to an ongoing fermentation process. This practice is evident in the MS fermentation during both seasons, where the addition of freshly harvested seeds can be detected by the presence of *T. cacao* chloroplast sequences in the 16S rRNA gene amplicon libraries. Under regular fermentation conditions, the cocoa seed dies and the detection of the chloroplast OTU decreases in abundance relative to the bacterial OTUs, however in the MS fermentation, chloroplast abundance increases at 36 h, accompanied by a second unexpected peaks of abundance of LAB which coincides with the idea of the addition of fresh material by the farmer, as seen in Figure S3.

### Microbial Composition Differs Between Surface and Middle Zone of the Bean Mass

The microbial composition was compared between the upper (surface) and middle zone (internal bean mass) of the wooden box, to explore how the exposition to external (environmental) conditions affects the dynamics of the microbial communities within the same fermentation process. The comparison was done by subtracting the middle zone relative abundance of each OTU from their abundance in the upper zone; values less than zero represented higher abundance in the middle zone, while values greater than zero indicated higher relative abundance in the upper zone.

The comparison between the relative abundance of upper and middle zone shows heterogeneity in the dynamics of the microbial communities in both farms (Figure S5). The relative abundance analysis shows that the LAB group appears faster and becomes more abundant in the middle zone than in the upper zone independently of the seasons and farm, during the first 36 h. In contrast, the ENT group has a higher abundance in the upper zone during the first 12–14 h. These results also show that the transition from LAB to AAB is faster in the upper layer. These patterns do not seem to be the result of differences in temperature as the analysis of temperature profiles between zones showed no significant difference (Figure S4), which suggest that the different environmental exposition (e.g., higher oxygen concentration) may be responsible for the variability in the microbial succession and hence on bean fermentation.

### Higher Resolution of Bacterial Oligotypes Identifies Dominant Variants

**Figure 2:**
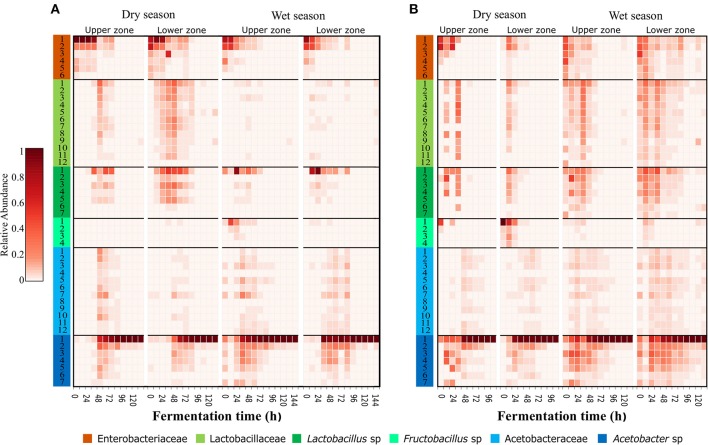
Dominant and transient oligotypes of bacteria in each process of cocoa bean fermentation for both agro-ecological zones. Heatmap of relative abundance of identified oligotypes, during both seasons and both sampling zones for Montaña Santandereana (MS) and Baja Cafetera (BC) **(A,B)**, respectively. Oligotypes are represented by rows, and the abundance is represented by the intensity of the box color. Each oligotype is colored at the leftmost column according to the original OTU taxonomical assignment (Figure 1) and the number corresponds to the oligotype number as depicted in Table S5.

The oligotyping analysis was used to detect informative variants for each bacterial OTU (Eren et al., 2013) and to establish its variation between locations, sampling seasons and along the fermentation process. The use of this method allowed the detection of 42–48 oligotypes in fermentation process monitoring (Table 1). From the 48 oligotypes detected in this study, a total of 30 oligotypes were identified for OTUs with the taxonomic assignment at the family level: 12 for the *Lactobacillaceae* OTU, 12 for *Acetobacteraceae* OTU, and 6 for *Enterobacteriaceae* OTU. In the case of OTUs assigned to genus 18 oligotypes were found; 7 assigned to the *Lactobacillus* sp. OTU, 7 to the *Acetobacter* sp. OTU, and 4 to the *Fructobacillus* sp. OTU (Figure 2 and Table S5). In general, the highest diversity of oligotypes was observed at the beginning of the transition between functional bacterial groups (ENT to LAB and LAB to AAB). The highest values of Shannon diversity were observed after 24 h where the LAB group was the most abundant group and the lowest was observed after 72 h where a few AAB oligotypes were dominant (Figure S6 and Table S5).

The specific analysis of oligotypes diversity and abundance within the different groups, ENT, LAB and AAB allowed us to identify dominant and secondary oligotypes. In general, the most abundant oligotypes of each group were present in both farms and both sampling seasons (Figure 2). In the case of the ENT group, the patterns of abundance and the level of dominance changed between farms, where the oligotype Enterobacteriaceae-1 was more abundant and dominant in BC, being 2–13 times more abundant than the Enterobacteriaceae-2 oligotype. In contrast, in MS there is higher evenness between the ENT oligotypes, where the most abundant oligotype throughout the fermentation was Enterobacteriaceae-2 oligotype being 1.2–1.4 times more abundant than Enterobacteriaceae-1. In the case of the LAB group, there was a higher variability of the most abundant variants in MS than in BC. In the former, the most abundant oligotype was Lactobacillus-1 being 3–147 times more abundant than the second most abundant oligotype from the same group (Table S5). In MS, Fructobacillus-1 was the most abundant LAB oligotype during May sampling, while in June there was a more even distribution of oligotypes and the most abundant one differed between upper and middle zones, being Lactobacillus-1 more abundant in the upper zone and Lactobacillus-2 the most abundant in the middle zone. For the AAB group, the same oligotype, Acetobacter-1, was found to be dominant for both farms and sampling times, being 24–127 times more abundant than the second most abundant oligotype from the same group.

### Relationship Between Bacterial Isolates and Oligotypes

**Figure 3:**
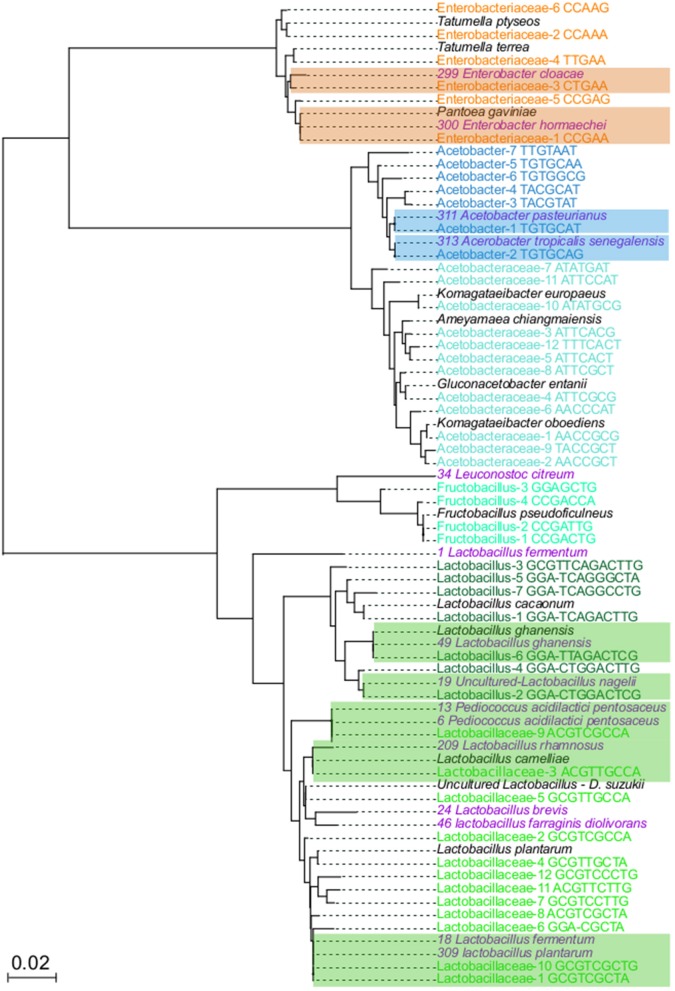
Neighbor joining tree to related 16S rRNA genes from variants and isolates. In total, 74 sequences were aligned with MUSCLE, using the default parameters Neighbor joining algorithm was used to elucidate the relationship between the sequences, using 1,000 boostrap. Colored names correspond to taxons from: *Enterobacteriaceae* (orange), *Acetobacteraceae* (light blue), Acetobacter (dark blue), Fructobacillus (light green), *Lactobacillaceae* (dark green), isolates (purple), and NR best hits (black). Highlighted branches correspond to the branches with close related oligotypes and bacterial isolates.

To demonstrate that oligotypes sequences represent real bacterial groups, isolation, and sequencing of the relevant bacterial groups were done. From each isolate, the 16S rRNA gene was sequenced and a phylogenetic tree was generated to compare the oligotypes with isolates and to reference sequences deposited in the public databases (Figure 3). The isolation and molecular identification show that three different functional bacterial groups were successfully recovered from the fermented beans. A total of 51 isolates were obtained, 16 colony morphotypes for AAB, 32 colony morphotypes for LAB and 2 colony morphotypes for ENT.

The phylogenetic tree of isolates and oligotypes show that isolation recovered close related organisms (sequences with <1% differences) at least 14 out of 48 oligotypes (Figure 3), these isolates represent the most dominant and abundant oligotypes of each group. For the ENT group, Enterobacteriaceae-1 and Enterobacteriaceae-3 oligotypes were phylogenetically closely related to *Pantoea gavinae* and *Enterobacter hormaechei*, respectively, while Enterobacteraceae-5 clustered with one isolate related to *Enterobacter cloacae*. For the LAB group, Lactobacillaceae-1, Lactobacillaceae-6, Lactobacillaceae-10 clustered with 7, 2 and 2 isolates, respectively, these isolates were more closely related to *Lactobacillus plantarum* and *L. fermentum*. Lactobacillaceae-3 clustered with one isolate closely related to *L. rhamnosus* and *L. cameliae*. Lactobacillaceae-9 clustered with 5 isolates, more closely related to *Pediococcus acidilactici* and *P. pentosaceus*. In the case of Lactobacillus-2 and Lactobacillus-4, one isolate cluster with each oligotype, and both were more closely related to *Lactobacillus nagelii*. Lactobacillus-6, clusters with one isolate more closely related to *Lactobacillus ghanensis*. Finally, Fructobacillus-3 clustered with one isolate related to *Leuconostoc citreum*. For the AAB group, Acetobacter-1 clustered with 10 isolates, closely related to *Acetobacter pasteurianus*. Acetobacter-2 clustered with 4 isolates, closely related to *Acetobacter tropicalis* and *A. senegalensis*

Around 34 oligotypes were not recovered with our in-*vitro* approach. In the case of ENT, oligotypes that did not have isolates were closely related to *Tatumella ptyseos* and *T. terrea*. One of the groups for which we did not recover any close related isolates was the *Acetobacteraceae*, phylogenetic analysis show that these oligotypes were closely related to sequences of the *Komagataeibacter oboediens, Komagataeibacter europaeus, Ameyamaea chiangmaiensis*, and *Gluconobacter entanii*. In the case of the LAB group, oligotypes with no isolates were related to *Lactobacillus cacaonum, Uncultured Lactobacillus* present in *Drosophila suzukii and Fructobacillus pseudoficulneus*. Finally, for 12 oligotypes we did not have a closely related species. Detailed description of phylogenetic relationships of oligotypes is presented in Table S6.

### The Patterns of Diversity, Dominance and Interaction of Oligotypes

**Figure 4:**
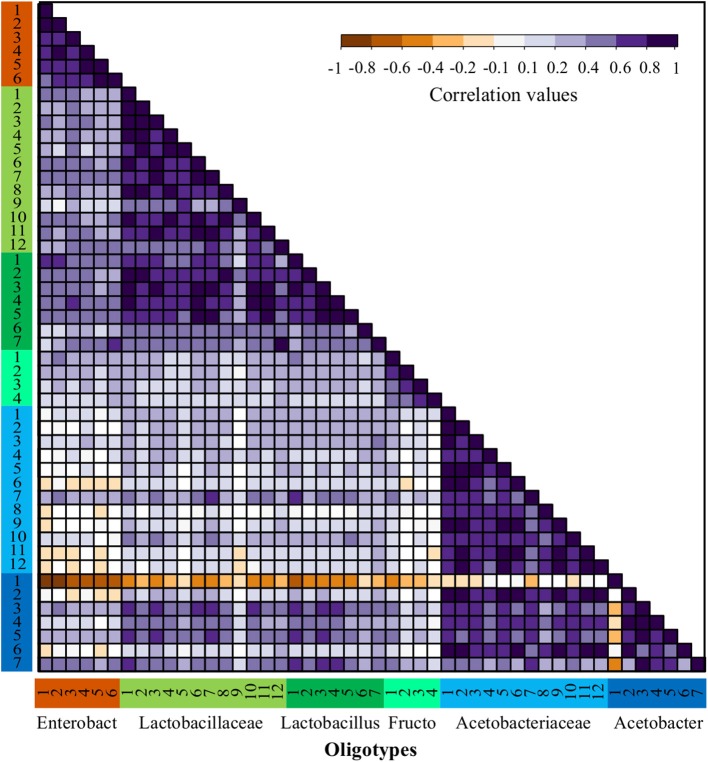
Abundance correlations between oligotypes from bacterial OTUs through all fermentations process. Spearman correlations matrix between all bacteria oligotypes detected in the full dataset including both agro-ecological zones. Oligotypes are sorted in the same order and colored by OTU as shown in Figure 2 and the number corresponds to the oligotype number as depicted in Table S5.

The inference of ecological interaction between oligotypes can be an informative criterion to select a starter culture and predict the outcome of the addition of specific isolates to a spontaneous cocoa fermentation system. Such interactions can be inferred using the relative abundances of the different populations through times and locations (Caporaso et al., 2011). Given that most oligotypes were present in both AEZ and that a future starter culture should be functional in different regions, we analyzed the abundance correlation (coexistence) between oligotypes in both AEZ and used this correlation matrix as a proxy to determine the positive, neutral, and negative correlations between the oligotypes (Figure 4).

The patterns of correlation reflecting possible positive interactions were observed more often among oligotypes obtained within similar functional groups (ENT, LAB, and AAB), however, this was not the case for all the functional groups. For instance, for the ENT there is a strong positive correlation between Enterobacteriaceae-1 and Enterobacteriaceae-2, which is consistent in all farms. Within the LAB functional group, the Lactobacillus and *Lactobacillaceae* oligotypes have a significant positive correlation. In contrast, a low correlation was observed between these two former groups and the *Fructobacillus* oligotypes. In the case of AAB, the *Acetobacteraceae* oligotypes show a strong positive correlation with most *Acetobacter* sp. oligotypes, nonetheless, there is a significant negative interaction between the most abundant oligotype from the *Acetobacter* sp., Acetobacter-1, with almost every other oligotype. This negative correlation can be observed in Figure 2 by its dominance in all fermentation processes at the last time points, where usually no other oligotype was observed.

The dominance indexes were also evaluated within each bacterial group (AAB, ENT, and LAB) to establish which groups might be better candidates for the isolation of starter cultures. We assumed that groups with high dominance indexes might be better for the isolation of highly abundant oligotypes and show a higher potential of success as we assured that the isolate should be highly competitive. To do this, we quantified the distribution of the dominance index within each group, the samples used for this quantification were the ones where the relative abundance of the group was higher than 10% to avoid stochastic effects (Figure S7). It is possible to observe a high dominance distribution for AAB with a very skewed distribution where the median of the dominance index is higher than 0.9, which agrees with the patterns of correlation observed for the Acetobacter-1 oligotype (see above). In the case of the ENT group, the distribution of the dominance index seems to be more intermediate with a median higher than 0.5, but with a bimodal distribution of the data with higher density around the dominance index of 0.5 and 0.8, this seems to be the results of the intermittent dominance of the Enterobacteriaceae-1 and Enterobacteriaceae-2 oligotypes. In the case of LAB, the violin plot shows a distribution skewed toward values of low dominance with a median lower than 0.3, which agrees with the high diversity observed for these functional groups and the lack of a clear dominant oligotype within the group throughout the experiment.

### Clustering Fermentation Processes by Geographic Location Using Correlation Matrices

A correlation matrix between oligotypes using the relative abundances of all samples was used to compare fermentation processes allowing us to control the effect of different speeds of fermentation and focus on the interaction of the bacterial communities. In total eight matrices were generated (as we had two farms, two seasons, and two depths). The correlation between matrices was calculated using a Mantel test (Sokal and Oden, 1978) implemented in PAST (Hammer et al., 2001), the values of correlation were used to cluster the fermentation processes and to calculate the Bray Curtis index for visualization using Principal Coordinate Analysis. The multivariate analyses of the correlation between all fermentation processes show a clear clustering by farm and season (Figure S8). The Principal Coordinate Analysis shows an 81% variation explained within the first two axes, 56 and 25% of the variability, respectively. An ordination plot of these axis shows that the first axis splits fermentation processes by farm, while a combination of the first and second axis cluster fermentations by season. Therefore, overall, the analysis shows the clustering of fermentations from the same farms and season.

## Discussion

### Microbial Community Analyses of Cocoa Fermentation Agro-Ecological Zones

This study examined in detail, in terms of sampling effort and taxonomic resolution, the bacterial and yeast species populations of the cacao fermentation process present in two independent agro-ecological zones and at two different seasons in Colombia. The bacterial and yeasts species found in both farms corresponded to those previously reported in fermentation process from Brazil (Papalexandratou et al., 2011b; Pereira et al., 2016), Ecuador (Papalexandratou et al., 2011a), Ghana (Garcia-Armisen et al., 2010), Indonesia (Ardhana and Fleet, 2003), Malaysia (Papalexandratou et al., 2013), and Mexico (Arana-Sánchez et al., 2015). Furthermore, the microbial composition and succession associated to the cocoa fermentation is in agreement with the reports obtained with methodologies such as PCR-DGGE and RFLP (Cocolin et al., 2013; Arana-Sánchez et al., 2015), in particular, identifying bacterial OTUs belonging to the *Enterobacteriaceae* family, *Lactobacillaceae* family, *Lactobacillus* sp., *Fructobacillus* sp., *Acetobacteraceae* family and *Acetobacter* sp., and yeast OTUs belonging to *Hanseniaspora opuntiae, Picha* sp., *Pichia kudriavzevi*, and *Wickerhamomyces pijperi*.

A closer look at the microbial diversity using the Minimum Entropy Decomposition (MED) (Eren et al., 2015), allowed us to identified and explore the pattern of dominance of bacterial finer groups in: (i) different times, (ii) zones of the fermentation box, (iii) time of the year, and (iv) agro-ecological zones. In contrast to the low OTU diversity, we observed high oligotype diversity (Figure 2) with characteristic patterns of variation as a function of time. Many of the detected oligotypes were recovered by culture-based methods supporting that these sequence variants are real and could be used to monitor fermentation processes. Is interesting that regardless that different locations and season were sampled, the overall composition of oligotypes was very similar between farms, suggesting that most of these microorganisms might be natural inhabitants of *T. cacao* either as endophytic or epiphytic microbiota.

In agreement with previous reports, the *Enterobacteriaceae* group was consistently detected in both farms during the early stages of cocoa bean fermentation, within this group we found oligotypes closely related to genus *Pantoea, Enterobacter*, and *Tatumella*, all of these genera have been previously reported in cocoa bean fermentation all over the world (Garcia-Armisen et al., 2010; Mota-Gutierrez et al., 2018; Papalexandratou et al., 2019; Serra et al., 2019). These bacteria are common inhabitants of the plant tissues (Papalexandratou et al., 2011c; Pereira et al., 2013a), which could explain their presence in the early stages of fermentation in both farms. For instance, some species such as *Enterobacter cloacae* has been described as part of healthy *T. cacao* plants and successfully colonize seedlings (Leite et al., 2013). Furthermore, studies in other fermented vegetable foods have identified similar patterns with Enterobacteriaceae being present in the early days of spontaneous carrot juice fermentation and Kimchi (Park et al., 2012; Wuyts et al., 2018), suggesting a close association with multiple plant tissues. The contribution of the Enterobacteriaceae to cocoa fermentation requires further characterization, however, the study in carrot fermentation (Wuyts et al., 2018) and our preliminary metatranscriptomic work (data not shown) suggests that the Enterobacteriaceae are metabolically active during early fermentation. Furthermore, a recent metagenomic analysis suggests that these bacteria might be relevant in fermentation and seem to contribute to having the capability to degrade pectin and assimilate citrate (Illeghems et al., 2015).

Also in agreement with the described successions, LAB increased their relative abundance during the anaerobic phase of the fermentation, initial 24–48 h. Compared to the other groups, the LAB group is the one with the highest OTU and oligotype diversity, representing 23 out of the 48 oligotypes detected for all bacterial groups, such diversity also seem to be highly homogeneous, as the quantification of the dominance index showed (Figure S7), the lowest values, suggesting coexistence of multiple species and possibly source partitioning during this stage of the fermentation. A better understanding of these patterns of diversity of species is necessary to establish the relationships between these fine level diversity and metabolic function. In fact, there is evidence that suggests that not all oligotypes or even strains have the same role and that close related organisms might differ in their metabolic contribution to the fermentation process. For instance, it was identified through metafluxome analysis that *Lactobacillus fermentum* strain NCC575 uses fructose as an alternative external electron acceptor, while strain NCC52 does not (Adler et al., 2013) and therefore the presence of multiple carbon sources may allow their coexistence.

The comparison between LAB isolates and oligotypes shows that the recover groups by both approaches are related to the ones previously described in cocoa fermentation. Phylogenetic analysis show that even though oligotypes provide a higher resolution that OTUs, more than one species could be closely related or even identical to a specific oligotype reflecting the limitations of the use of 16S rRNA as a molecular marker. In terms of concurrence within the LAB group the *Lactobacillus* and *Lactobacillaceae* oligotypes overlap with some differences in dominance and relative abundance, such pattern in sympatry might reflect resource partitioning and micro-niche adaptation of the oligotypes (Schloss et al., 2009). A different case is observed between these two former groups and the *Fructobacillus* oligotypes, whenever the *Fructobacillus* oligotypes were abundant the *Lactobacillus* and *Lactobacillaceae* were not, showing low correlation and possibly a pattern of competitive exclusion between these groups (Ouattara et al., 2017). The source of inoculation of LAB in the cocoa fermentation is not clear, but several studies suggest that these bacteria might be associated with the pods or tree leaves. Recent work in *Origanum vulgare* L (oregano) has shown that *Lactobacillus plantarum*, commonly found in cocoa seed fermentation, is a common inhabitant of leaves of other plants dominating the microbial community either as an epiphyte or endophyte (Pontonio et al., 2018). This could be the case of many other LAB, such as *Lactobacillus cacaonum* that was first isolated from fermented cocoa beans (De Bruyne et al., 2009), but that might actually be a common inhabitant of the plant.

The Acetic Acid Bacteria group became more abundant and dominant toward the end of fermentation as a result of cocoa bean mixing. A total of 17 AAB oligotypes were detected, with a higher number of oligotypes observed at the transition point between anaerobic to oxygenic conditions, that correspond to the ecological succession from LAB to AAB (see the Shannon indexes, Figure S6), suggesting that at the beginning of the succession there are more niches for different AAB oligotypes to colonize, with a very fast decline on the number of AAB oligotypes, with Acetobacter-1, becoming the most abundant and dominant oligotype. This was observed in all fermentation processes evaluated despite the geographic location of the farm or the season of sampling. These results agree with previous work that shows that *A. pasteurianus*, the most closely related to Acetobacter-1 oligotype, has a higher prevalence than other Acetobacter species (Ardhana and Fleet, 2003). Furthermore, functional characterization of oxidation of metabolites present in cocoa fermentation shows that *A. pasteurianus* might be a better competitor than *A. senegalensis* and *A. fabarum*, due to faster oxidation of ethanol into acetic acid coupled to fast oxidation of lactic acid into acetoin (Moens et al., 2014). Both metabolic capabilities generate electrons that enter the respiratory chain generating energy and therefore are an adaptive advantage that favors the dominance of *A. pasteruranis*. The presence of other Acetobacter species at the beginning of the fermentation could be associated with the availability of higher diversity of nutrients. In fact, comparative genome analysis *of A. ghanensis* and *A. senegalensis* (more closely related to Acerobacter-2 oligotype) shows the dependence of these two species on mannitol, glycerol, and lactate (Illeghems et al., 2016). Additionally, this work also reported that these two species might have a less efficient acid stress response that *A. pasteruranis*. Although interaction and competition might be happening between oligotypes within groups (ENT, LAB, and AAB), almost no influence between oligotypes from different groups was observed.

### Variability on the Microbial Dynamics During Cocoa Fermentation

The use of sequencing of genetic markers not only allowed us to identify taxonomically the microbiota associated with cocoa bean fermentation but also provided a tool to monitor the variability of the process. Here we showed that artisanal open wooden fermenters have and inherent heterogeneity that affects the abundance and transition of the three bacterial groups in the upper and middle zone of the bean mass. The lower abundance of LAB in the upper zone is likely the consequence of a higher oxygen concentration, that might prevent the efficient colonization of LAB in the most external cocoa bean layers, due to lower oxygen tolerance (Cesselin et al., 2011), higher oxygen can also favor acetic acid production by AAB, generating adverse condition for LAB to colonize. This heterogeneity within the same bean mass, have important implication for the establishment of protocol or fermentation technologies as artisanal fermenters where environmental conditions are difficult to control might inevitably result in different rates of fermentation of the beans. In this sense, farmers' decision of when and how often to mix the bean mass has a significant effect in bean fermentation heterogeneity. The use of phylogenetic markers also allowed us to identify undesirable local practices such as the addition of fresh collected beans into an ongoing fermentation process (Figure S3). Such practices alter the dynamic of the microbial fermentation, by providing fresh pulp to the fermentation LAB become dominant in the fermentation affecting the transition to AAB and therefore the production of acetic acid. Uneven fermentation of beans in terms of time affects the final quality of the fermented beans, resulting in a proportion of the beans ending as either under or over fermented. The reason for such practices seems to mostly relate to the cost of harvesting labor and the uneven ripening of pods in the plantation. These results show that the use of culture-independent methods is a powerful tool that asses the microbial dynamics and therefore could be used to generate an informed decision to be able to standardize the fermentation process and follow up on the effects of implemented protocol modifications.

Finally, fermentation processes are not entirely homogenous across regions, in some cases the transition of the ecological successions happened faster, and in consequence, some fermentation ended earlier than others, these differences difficult a direct time-point to time-point comparison between locations. Here we show that co-abundance matrices between oligotypes can be used to solve this issue by comparing instead the interaction between the bacterial populations. This showed a clear clustering of samples by the farm, reflecting either the local conditions effect (e.g., temperature, humidity) or the effect of the different fermentation protocols used by the farmers. In any case, microbial interactions derived from patterns of oligotype co-abundance seem to be specific and therefore reflecting a strong relationship with location.

## Conclusions

This is the first study on the microbiome dynamics of cacao fermentation that is performed at a high level of resolution in terms of sampling times (every 12 h for up to 10 days) and taxonomic resolution, using deep marker gene (16S rRNA gene and ITS) sequencing and oligotype analysis. Two main contrasting observations were observed, first, at a coarse level, the fermentation process is extremely conserved with the same functional groups, and even dominant oligotypes, being detected in all the different fermentations analyzed, with predictable successions over time. Second, at a fine level, heterogeneity of the process is observed in terms of the exact time of microbial transitions and oligotype dominance and diversity, dependent on specific environmental conditions such as oxygen availability and addition of fresh material. Given the results, it suggests that following studies should focus on analyzing the microbiota associated with the fermentation at high resolution since those subtle changes may have a significant impact on the final product.

Microbial ecology studies in the food industry have the potential to guide better decision making toward the selection of microbial consortia for specific tasks. In our case, the dominance and co-abundance matrices allowed us to identified dominant populations within OTUs, for selection as starter cultures and for monitoring the fermentation process. The oligotype detection can be implemented to select, monitor and validate the inoculation of specific microorganisms to modulate and improve chocolate quality. Some of the microorganisms we observed in our study have been suggest as microbial starters (Schwan, 1998; Papalexandratou et al., 2013; Magalhães da Veiga Moreira et al., 2017), such as *Acetobacter pasteurianus* and *Lactobacillus fermentum* (Papalexandratou et al., 2013; Illeghems et al., 2016), additionally, some of the yeasts groups (*Hanseniaspora opuntiae* and *Picha* sp.) have been reported in the fermentation process from other countries (Schwan et al., 2014) and some have been used as culture starter (Batista et al., 2016). The dominance patterns suggest that to design starter cultures, only a few isolates of each group might be necessary; however, experiments are needed to elucidate the importance of non-dominant bacteria in the fermentation and the generation of chemical precursors of cocoa quality Future perspectives, will direct these studies toward the design of family-specific molecular markers, e.g., *phyloTAGs* (Caro-Quintero and Howard, 2015), that provide higher resolution than 16S rRNA gene allowing monitoring of starter cultures in controlled and spontaneous conditions, and to understand the mechanisms for higher dominance of specific populations.

## Data Availability Statement

The bacterial and fungal sequence data generated in this study using MiSeq have been deposited and are available in the NCBI Sequence Read Archive (SRA) under BioProject PRJNA492720. The 16S rRNA sanger sequences of isolates and oligotypes are provided as Supplementary Information.

## Author's Note

This manuscript has been released as a pre-print at BioRxiv, MP-M (Pacheco-Montealegre et al., 2019). Sampling was conducted under the guidelines indicated in resolution 1466 of December 03, 2014 of ANLA, Colombia.

## Author Contributions

MP-M performed all the data, computational analyses and statistics, discussed the results, and wrote the manuscript. LD-M and LB-R performed the sample collection, amplicon metagenomic sequencing, and participated on writing of the manuscript. AR mentored on bioinformatics analysis, participated on the discussion, and writing of the manuscript. AC-Q did the experimental designed, participated in the sample collection, and advised on bioinformatic analyses, results discussion, and writing of the manuscript.

### Conflict of Interest

The authors declare that the research was conducted in the absence of any commercial or financial relationships that could be construed as a potential conflict of interest.
