# Supplementary material for: Fine Resolution Analysis of Microbial Communities Provides Insights Into the Variability of Cocoa Bean Fermentation
Source: Front Microbiol. 2020 Apr 15;11:650. doi: 10.3389/fmicb.2020.00650 (PMC7174660; doi:10.3389/fmicb.2020.00650)
Supplement: Supplementary file 1 [file Data_Sheet_1.PDF]

Fine Resolution Analysis of Microbial Communities Provide Insights into the Variability of Cocoa Bean Fermentation

M. E. Pacheco-Montealegre1,2, L. L. Dávila-Mora1, L. M. Botero-Rute1, A. Reyes2 and A. Caro-Quintero1*

1 Centro de Investigación Agropecuaria AGROSAVIA sede Tibaitatá, Mosquera, Colombia.
2 Grupo de Biología Computacional y Ecología Microbiana BCEM - Max Planck Tandem Group in Computational Biology, Universidad de los Andes, Bogotá, Colombia

FASTA Sequences of oligotypes and isolates, The "*" in the name was used to identify those sequences used in Figure 3

```
>Enterobacteriaceae-1_CCGAA*
GTGCAAGCGTTAATCGGAATTACTGGGCGTAAAGCGCACGCAGGCGGTCTGTCAAGTCGG
ATGTGAAATCCCCGGGCTCAACCCGGGAACTGCATTCGAAACTGGCAGGCTAGAGTCTTG
TAGAGGGGGGTAGAATTCCAGGTGTAGCGGTGAAATGCGTAGAGATCTGGAGGAATACCG
GTGGCGAAGGCGGCCCCCTGGACAAAGACTGACGCTCAGGTGCGAAAGCGTGGGGAGC
>Enterobacteriaceae-2_CCAAA*
GTGCAAGCGTTAATCGGAATTACTGGGCGTAAAGCGCACGCAGGCGGTCTGTCAAGTCGG
ATGTGAAATCCCCGGGCTCAACCCGGGAACTGCATTCGAAACTGGCAGACTAGAGTCTTG
TAGAGGGGGGTAGAATTCCAGGTGTAGCGGTGAAATGCGTAGAGATCTGGAGGAATACCG
GTGGCGAAGGCGGCCCCCTGGACAAAGACTGACGCTCAGGTGCGAAAGCGTGGGGAGC
>Enterobacteriaceae-3_CTGAA*
GTGCAAGCGTTAATCGGAATTACTGGGCGTAAAGCGCACGCAGGCGGTCTGTCAAGTCGG
ATGTGAAATCCCCGGGCTCAACCTGGGAACTGCATTCGAAACTGGCAGGCTAGAGTCTTG
TAGAGGGGGGTAGAATTCCAGGTGTAGCGGTGAAATGCGTAGAGATCTGGAGGAATACCG
GTGGCGAAGGCGGCCCCCTGGACAAAGACTGACGCTCAGGTGCGAAAGCGTGGGGAGC
>Enterobacteriaceae-4_TTGAA*
GTGCAAGCGTTAATCGGAATTACTGGGCGTAAAGCGCACGCAGGCGGTCTGTCAAGTCGG
ATGTGAAATCCCCGGGCTTAACCTGGGAACTGCATTCGAAACTGGCAGGCTAGAGTCTTG
TAGAGGGGGGTAGAATTCCAGGTGTAGCGGTGAAATGCGTAGAGATCTGGAGGAATACCG
GTGGCGAAGGCGGCCCCCTGGACAAAGACTGACGCTCAGGTGCGAAAGCGTGGGGAGC
>Enterobacteriaceae-5_CCGAG*
GTGCAAGCGTTAATCGGAATTACTGGGCGTAAAGCGCACGCAGGCGGTCTGTCAAGTCGG
ATGTGAAATCCCCGGGCTCAACCCGGGAACTGCATTCGAAACTGGCAGGCTAGAGTCTTG
TAGAGGGGGGTAGAATTCCAGGTGTAGCGGTGAAATGCGTAGAGATCTGGAGGAATACCG
GTGGCGAAGGCGGCCCCCTGGACGAAGACTGACGCTCAGGTGCGAAAGCGTGGGGAGC
>Enterobacteriaceae-6_CCAAG*
GTGCAAGCGTTAATCGGAATTACTGGGCGTAAAGCGCACGCAGGCGGTCTGTCAAGTCGG
ATGTGAAATCCCCGGGCTCAACCCGGGAACTGCATTCGAAACTGGCAGACTAGAGTCTTG
TAGAGGGGGGTAGAATTCCAGGTGTAGCGGTGAAATGCGTAGAGATCTGGAGGAATACCG
GTGGCGAAGGCGGCCCCCTGGACGAAGACTGACGCTCAGGTGCGAAAGCGTGGGGAGC
>Lactobacillaceae-1_GCGTCGCTA*
TGGCAAGCGTTGTCCGGATTTATTGGGCGTAAAGCGAGCGCAGGCGGTTTTTTAAGTCTG
ATGTGAAAGCCTTCGGCTCAACCGAAGAAGTGCATCGGAAACTGGGAAACTTGAGTGCAG
AAGAGGACAGTGGAACTCCATGTGTAGCGGTGAAATGCGTAGATATATGGAAGAACACCA
GTGGCGAAGGCGGCTGTCTGGTCTGTAACTGACGCTGAGGCTCGAAAGTATGGGTAGC
```

>Lactobacillaceae-2_GCGTCGCCA*
TGGCAAGCGTTGTCCGGATTTATTGGGCGTAAAGCGAGCGCAGGCGGTTTTTTAAGTCTG
ATGTGAAAGCCTTCGGCTCAACCGAAGAAGTGCATCGGAAACTGGGAAACTTGAGTGCAG
AAGAGGACAGTGGAACTCCATGTGTAGCGGTGAAATGCGTAGATATATGGAAGAACACCA
GTGGCGAAGGCGGCTGTCTGGTCTGTAACTGACGCTGAGGCTCGAAAGCATGGGTAGC
>Lactobacillaceae-3_ACGTTGCCA*
TGGCAAGCGTTATCCGGATTTATTGGGCGTAAAGCGAGCGCAGGCGGTTTTTTAAGTCTG
ATGTGAAAGCCTTCGGCTTAACCGAAGAAGTGCATCGGAAACTGGGAAACTTGAGTGCAG
AAGAGGACAGTGGAACTCCATGTGTAGCGGTGAAATGCGTAGATATATGGAAGAACACCA
GTGGCGAAGGCGGCTGTCTGGTCTGTAACTGACGCTGAGGCTCGAAAGCATGGGTAGC
>Lactobacillaceae-4_GCGTTGCTA*
TGGCAAGCGTTGTCCGGATTTATTGGGCGTAAAGCGAGCGCAGGCGGTTTTTTAAGTCTG
ATGTGAAAGCCTTCGGCTTAACCGAAGAAGTGCATCGGAAACTGGGAAACTTGAGTGCAG
AAGAGGACAGTGGAACTCCATGTGTAGCGGTGAAATGCGTAGATATATGGAAGAACACCA
GTGGCGAAGGCGGCTGTCTGGTCTGTAACTGACGCTGAGGCTCGAAAGTATGGGTAGC
>Lactobacillaceae-5_GCGTTGCCA*
TGGCAAGCGTTGTCCGGATTTATTGGGCGTAAAGCGAGCGCAGGCGGTTTTTTAAGTCTG
ATGTGAAAGCCTTCGGCTTAACCGAAGAAGTGCATCGGAAACTGGGAAACTTGAGTGCAG
AAGAGGACAGTGGAACTCCATGTGTAGCGGTGAAATGCGTAGATATATGGAAGAACACCA
GTGGCGAAGGCGGCTGTCTGGTCTGTAACTGACGCTGAGGCTCGAAAGCATGGGTAGC
>Lactobacillaceae-6_GGA-CGCTA*
TGGCAAGCGTTGTCCGGATTTATTGGGCGTAAAGGGAACGCAGGCGGTTTTTAAGTCTGA
TGTGAAAGCCTTCGGCTCAACCGAAGAAGTGCATCGGAAACTGGGAAACTTGAGTGCAGA
AGAGGACAGTGGAACTCCATGTGTAGCGGTGAAATGCGTAGATATATGGAAGAACACCAG
TGGCGAAGGCGGCTGTCTGGTCTGTAACTGACGCTGAGGCTCGAAAGTATGGGTAGC
>Lactobacillaceae-7_GCGTCCTTG*
TGGCAAGCGTTGTCCGGATTTATTGGGCGTAAAGCGAGCGCAGGCGGTTTTTTAAGTCTG
ATGTGAAAGCCTTCGGCTCAACCGAAGAAGTGCATCGGAAACTGGGAAACTTGAGTGCAG
AAGAGGACAGTGGAACTCCATGTGTAGCGGTGAAATGCGTAGATATATGGAAGAACACCA
GTGGCGAAAGCGGCTCTCTGGTCTGTAACTGACGCTGAGGTTCGAAAGTGTGGGTAGC
>Lactobacillaceae-8_ACGTCGCTA*
TGGCAAGCGTTATCCGGATTTATTGGGCGTAAAGCGAGCGCAGGCGGTTTTTTAAGTCTG
ATGTGAAAGCCTTCGGCTCAACCGAAGAAGTGCATCGGAAACTGGGAAACTTGAGTGCAG
AAGAGGACAGTGGAACTCCATGTGTAGCGGTGAAATGCGTAGATATATGGAAGAACACCA
GTGGCGAAGGCGGCTGTCTGGTCTGTAACTGACGCTGAGGCTCGAAAGTATGGGTAGC
>Lactobacillaceae-9_ACGTCGCCA*
TGGCAAGCGTTATCCGGATTTATTGGGCGTAAAGCGAGCGCAGGCGGTCTTTAAGTCTAA
TGTGAAAGCCTTCGGCTCAACCGAAGAAGTGCATTGGAAACTGGGAGACTTGAGTGCAGA
AGAGGACAGTGGAACTCCATGTGTAGCGGTGAAATGCGTAGATATATGGAAGAACACCAG
TGGCGAAGGCGGCTGTCTGGTCTGTAACTGACGCTGAGGCTCGAAAGCATGGGTAGC
>Lactobacillaceae-10_GCGTCGCTG*
TGGCAAGCGTTGTCCGGATTTATTGGGCGTAAAGCGAGCGCAGGCGGTTTTTTAAGTCTG
ATGTGAAAGCCTTCGGCTCAACCGAAGAAGTGCATCGGAAACTGGGAAACTTGAGTGCAG
AAGAGGACAGTGGAACTCCATGTGTAGCGGTGAAATGCGTAGATATATGGAAGAACACCA
GTGGCGAAGGCGGCTGTCTGGTCTGTAACTGACGCTGAGGCTCGAAAGTGTGGGTAGC
>Lactobacillaceae-11_ACGTTCTTG*
TGGCAAGCGTTATCCGGATTTATTGGGCGTAAAGCGAGCGCAGGCGGTTTTTTAAGTCTG
ATGTGAAAGCCTTCGGCTTAACCGAAGAAGTGCATCGGAAACTGGGAAACTTGAGTGCAG
AAGAGGACAGTGGAACTCCATGTGTAGCGGTGAAATGCGTAGATATATGGAAGAACACCA
GTGGCGAAAGCGGCTCTCTGGTCTGTAACTGACGCTGAGGTTCGAAAGTGTGGGTAGC
>Lactobacillaceae-12_GCGTCCCTG*
TGGCAAGCGTTGTCCGGATTTATTGGGCGTAAAGCGAGCGCAGGCGGTTTTTTAAGTCTG
ATGTGAAAGCCTTCGGCTCAACCGAAGAAGTGCATCGGAAACTGGGAAACTTGAGTGCAG
AAGAGGACAGTGGAACTCCATGTGTAGCGGTGAAATGCGTAGATATATGGAAGAACACCA

GAGGCGAAGGCGGCTCTCTGGACTGTAACTGACGCTGAGGCTCGAAAGTGTGGGTAGC
>Lactobacillus-1_GGA-TCAGACTTG*
TGGCAAGCGTTGTCCGGATTTATTGGGCGTAAAGGGAACGCAGGCGGTTTTTAAGTCTGA
TGTGAAAGCCTTCGGCTCAACCGAAGTCGTGCATTGGAAACTGGAAGACTTGAGTGCAGA
AGAGGAGAGTGGAACTCCATGTGTAGCGGTGAAATGCGTAGATATATGGAAGAACACCAG
TGGCGAAAGCGGCTCTCTGGTCTGTAACTGACGCTGAGGTTCGAAAGTGTGGGTAGC
>Lactobacillus-2_GGA-CTGGACTCG*
TGGCAAGCGTTGTCCGGATTTATTGGGCGTAAAGGGAACGCAGGCGGTCTTTAAGTCTGA
TGTGAAAGCCTTCGGCTTAACCGAAGTCGTGCATTGGAAACTGGGAGACTTGAGTGCAGA
AGAGGAGAGTGGAACTCCATGTGTAGCGGTGAAATGCGTAGATATATGGAAGAACACCAG
TGGCGAAAGCGGCTCTCTGGTCTGTAACTGACGCTGAGGTTCGAAAGCGTGGGTAGC
>Lactobacillus-3_GCGTTCAGACTTG*
TGGCAAGCGTTGTCCGGATTTATTGGGCGTAAAGCGAGCGCAGGCGGTTTTTTAAGTCTG
ATGTGAAAGCCTTCGGCTCAACCGAAGTCGTGCATTGGAAACTGGAAGACTTGAGTGCAG
AAGAGGAGAGTGGAACTCCATGTGTAGCGGTGAAATGCGTAGATATATGGAAGAACACCA
GTGGCGAAAGCGGCTCTCTGGTCTGTAACTGACGCTGAGGTTCGAAAGTGTGGGTAGC
>Lactobacillus-4_GGA-CTGGACTTG*
TGGCAAGCGTTGTCCGGATTTATTGGGCGTAAAGGGAACGCAGGCGGTCTTTAAGTCTGA
TGTGAAAGCCTTCGGCTTAACCGAAGTCGTGCATTGGAAACTGGGAGACTTGAGTGCAGA
AGAGGAGAGTGGAACTCCATGTGTAGCGGTGAAATGCGTAGATATATGGAAGAACACCAG
TGGCGAAAGCGGCTCTCTGGTCTGTAACTGACGCTGAGGTTCGAAAGTGTGGGTAGC
>Lactobacillus-5_GGA-TCAGGGCTA*
TGGCAAGCGTTGTCCGGATTTATTGGGCGTAAAGGGAACGCAGGCGGTTTTTAAGTCTGA
TGTGAAAGCCTTCGGCTCAACCGAAGTCGTGCATTGGAAACTGGAAGACTTGAGTGCAGA
AGAGGAGAGTGGAACTCCATGTGTAGCGGTGAAATGCGTAGATATATGGAAGAACACCAG
TGGCGAAGGCGGCTGTCTGGTCTGTAACTGACGCTGAGGCTCGAAAGTATGGGTAGC
>Lactobacillus-6_GGA-TTAGACTCG*
TGGCAAGCGTTGTCCGGATTTATTGGGCGTAAAGGGAACGCAGGCGGTTTTTAAGTCTGA
TGTGAAAGCCTTCGGCTTAACCGAAGTCGTGCATTGGAAACTGGAGAACTTGAGTGCAGA
AGAGGAGAGTGGAACTCCATGTGTAGCGGTGAAATGCGTAGATATATGGAAGAACACCAG
TGGCGAAAGCGGCTCTCTGGTCTGTAACTGACGCTGAGGTTCGAAAGCGTGGGTAGC
>Lactobacillus-7_GGA-TCAGGCCTG*
TGGCAAGCGTTGTCCGGATTTATTGGGCGTAAAGGGAACGCAGGCGGTTTTTAAGTCTGA
TGTGAAAGCCTTCGGCTCAACCGAAGTCGTGCATTGGAAACTGGAAGACTTGAGTGCAGA
AGAGGAGAGTGGAACTCCATGTGTAGCGGTGAAATGCGTAGATATATGGAAGAACACCAG
AGGCGAAGGCGGCTCTCTGGACTGTAACTGACGCTGAGGCTCGAAAGTGTGGGTAGC
>Fructobacillus-1_CCGACTG*
TCCCGAGCGTTATCCGGATTTATTGGGCGTAAAGCGAGCGCAGACGGTTTGATAAGTCTG
AAGTGAAAGCCCACAGCTCAACTGTGGAAGTGCTTTGGAAACTGTCAAACTTGAGTGCAG
TAGAGGTAAGTGGAACTCCATGTGTAGCGGTGGAATGCGTAGATATATGGAAGAACACCA
GTGGCGAAGGCGGCTTACTGGACTGCAACTGACGTTGAGGCTCGAAAGTGTGGGTAG
>Fructobacillus-2_CCGATTG*
TCCCGAGCGTTATCCGGATTTATTGGGCGTAAAGCGAGCGCAGACGGTTTGATAAGTCTG
AAGTGAAAGCCCACAGCTCAACTGTGGAAGTGCTTTGGAAACTGTCAAACTTGAGTGCAG
TAGAGGTAAGTGGAACTCCATGTGTAGCGGTGGAATGCGTAGATATATGGAAGAACACCA
GTGGCGAAGGCGGCTTACTGGACTGTAACTGACGTTGAGGCTCGAAAGTGTGGGTAG
>Fructobacillus-3_GGAGCTG*
TGGCAAGCGTTGTCCGGATTTATTGGGCGTAAAGCGAGCGCAGACGGTTTGATAAGTCTG
AAGTGAAAGCCCACAGCTCAACTGTGGAAGTGCTTTGGAAACTGTCAAACTTGAGTGCAG
TAGAGGTAAGTGGAACTCCATGTGTAGCGGTGGAATGCGTAGATATATGGAAGAACACCA
GTGGCGAAGGCGGCTTACTGGACTGCAACTGACGTTGAGGCTCGAAAGTGTGGGTAG
>Fructobacillus-4_CCGACCA*
TCCCGAGCGTTATCCGGATTTATTGGGCGTAAAGCGAGCGCAGACGGTTTGATAAGTCTG
AAGTGAAAGCCCACAGCTCAACTGTGGAAGTGCTTTGGAAACTGTCAAACTTGAGTGCAG

TAGAGGTAAGTGGAACTCCATGTGTAGCGGTGGAATGCGTAGATATATGGAAGAACACCA
GTGGCGAAGGCGGCTTACTGGACTGCAACTGACGTTGAGGCTCGAAAGCATGGGTAG
>Acetobacteraceae-1_AACCGCG*
GGGCAAGCGTTGCTCGGAATGACTGGGCGTAAAGGGCGCGTAGGCGGTTGACACAGTCAG
ATGTGAAATTCCCGGGCTTAACCTGGGGGCTGCATTTGATACGTGGCGACTAGAGTGTGA
GAGAGGGTTGTGGAATTCCCAGTGTAGAGGTGAAATTCGTAGATATTGGGAAGAACACCG
GTGGCGAAGGCGGCAACCTGGCTCATGACTGACGCTGAGGCGCGAAAGCGTGGGGAGC
>Acetobacteraceae-2_AACCGCT*
GGGCAAGCGTTGCTCGGAATGACTGGGCGTAAAGGGCGCGTAGGCGGTTGACACAGTCAG
ATGTGAAATTCCCGGGCTTAACCTGGGGGCTGCATTTGATACGTGGCGACTAGAGTGTGA
GAGAGGGTTGTGGAATTCCCAGTGTAGAGGTGAAATTCGTAGATATTGGGAAGAACACCG
GTGGCGAAGGCGGCAACCTGGCTCATTACTGACGCTGAGGCGCGAAAGCGTGGGGAGC
>Acetobacteraceae-3_ATTCACG*
GGGCAAGCGTTGCTCGGAATGACTGGGCGTAAAGGGCGCGTAGGCGGTTGTTACAGTCAG
ATGTGAAATTCCCGGGCTTAACCTGGGGGCTGCATTTGATACGTGACGACTAGAGTGTGA
GAGAGGGTTGTGGAATTCCCAGTGTAGAGGTGAAATTCGTAGATATTGGGAAGAACACCG
GTGGCGAAGGCGGCAACCTGGCTCATGACTGACGCTGAGGCGCGAAAGCGTGGGGAGC
>Acetobacteraceae-4_ATTCGCG*
GGGCAAGCGTTGCTCGGAATGACTGGGCGTAAAGGGCGCGTAGGCGGTTGTTACAGTCAG
ATGTGAAATTCCCGGGCTTAACCTGGGGGCTGCATTTGATACGTGGCGACTAGAGTGTGA
GAGAGGGTTGTGGAATTCCCAGTGTAGAGGTGAAATTCGTAGATATTGGGAAGAACACCG
GTGGCGAAGGCGGCAACCTGGCTCATGACTGACGCTGAGGCGCGAAAGCGTGGGGAGC
>Acetobacteraceae-5_ATTCACT*
GGGCAAGCGTTGCTCGGAATGACTGGGCGTAAAGGGCGCGTAGGCGGTTGTTACAGTCAG
ATGTGAAATTCCCGGGCTTAACCTGGGGGCTGCATTTGATACGTGACGACTAGAGTGTGA
GAGAGGGTTGTGGAATTCCCAGTGTAGAGGTGAAATTCGTAGATATTGGGAAGAACACCG
GTGGCGAAGGCGGCAACCTGGCTCATTACTGACGCTGAGGCGCGAAAGCGTGGGGAGC
>Acetobacteraceae-6_AACCCAT*
GGGCAAGCGTTGCTCGGAATGACTGGGCGTAAAGGGCGCGTAGGCGGTTGACACAGTCAG
ATGTGAAATTCCCGGGCTTAACCTGGGGGCTGCATTTGATACGTGCAGACTAGAGTGTGA
GAGAGGGTTGTGGAATTCCCAGTGTAGAGGTGAAATTCGTAGATATTGGGAAGAACACCG
GTGGCGAAGGCGGCAACCTGGCTCATTACTGACGCTGAGGCGCGAAAGCGTGGGGAGC
>Acetobacteraceae-7_ATATGAT*
GGGCAAGCGTTGCTCGGAATGACTGGGCGTAAAGGGCGCGTAGGCGGTTTTAACAGTCAG
ATGTGAAATTCCTGGGCTTAACCTGGGGGCTGCATTTGATACGTTGAGACTAGAGTGTGA
GAGAGGGTTGTGGAATTCCCAGTGTAGAGGTGAAATTCGTAGATATTGGGAAGAACACCG
GTGGCGAAGGCGGCAACCTGGCTCATTACTGACGCTGAGGCGCGAAAGCGTGGGGAGC
>Acetobacteraceae-8_ATTCGCT*
GGGCAAGCGTTGCTCGGAATGACTGGGCGTAAAGGGCGCGTAGGCGGTTGTTACAGTCAG
ATGTGAAATTCCCGGGCTTAACCTGGGGGCTGCATTTGATACGTGGCGACTAGAGTGTGA
GAGAGGGTTGTGGAATTCCCAGTGTAGAGGTGAAATTCGTAGATATTGGGAAGAACACCG
GTGGCGAAGGCGGCAACCTGGCTCATTACTGACGCTGAGGCGCGAAAGCGTGGGGAGC
>Acetobacteraceae-9_TACCGCT*
GGGCTAGCGTTGCTCGGAATGACTGGGCGTAAAGGGCGCGTAGGCGGTTGACACAGTCAG
ATGTGAAATTCCCGGGCTTAACCTGGGGGCTGCATTTGATACGTGGCGACTAGAGTGTGA
GAGAGGGTTGTGGAATTCCCAGTGTAGAGGTGAAATTCGTAGATATTGGGAAGAACACCG
GTGGCGAAGGCGGCAACCTGGCTCATTACTGACGCTGAGGCGCGAAAGCGTGGGGAGC
>Acetobacteraceae-10_ATATGCG*
GGGCAAGCGTTGCTCGGAATGACTGGGCGTAAAGGGCGCGTAGGCGGTTGTAACAGTCAG
ATGTGAAATTCCTGGGCTTAACCTGGGGGCTGCATTTGATACGTTGCGACTAGAGTGTGA
GAGAGGGTTGTGGAATTCCCAGTGTAGAGGTGAAATTCGTAGATATTGGGAAGAACACCG
GTGGCGAAGGCGGCAACCTGGCTCATGACTGACGCTGAGGCGCGAAAGCGTGGGGAGC
>Acetobacteraceae-11_ATTCCAT*
GGGCAAGCGTTGCTCGGAATGACTGGGCGTAAAGGGCGCGTAGGCGGTTGTTACAGTCAG

ATGTGAAATTCCCGGGCTTAACCTGGGGGCTGCATTTGATACGTGCAGACTAGAGTGTGA
GAGAGGGTTGTGGAATTCCCAGTGTAGAGGTGAAATTCGTAGATATTGGGAAGAACACCG
GTGGCGAAGGCGGCAACCTGGCTCATTACTGACGCTGAGGCGCGAAAGCGTGGGGAGC
>Acetobacteraceae-12_TTTCACT*
GGGCTAGCGTTGCTCGGAATGACTGGGCGTAAAGGGCGCGTAGGCGGTTGTTACAGTCAG
ATGTGAAATTCCCGGGCTTAACCTGGGGGCTGCATTTGATACGTGACGACTAGAGTGTGA
GAGAGGGTTGTGGAATTCCCAGTGTAGAGGTGAAATTCGTAGATATTGGGAAGAACACCG
GTGGCGAAGGCGGCAACCTGGCTCATTACTGACGCTGAGGCGCGAAAGCGTGGGGAGC
>Acetobacter-1_TGTGCAT*
GGGCTAGCGTTGCTCGGAATGACTGGGCGTAAAGGGCGTGTAGGCGGTTTGTACAGTCAG
ATGTGAAATCCCCGGGCTTAACCTGGGAGCTGCATTTGATACGTGCAGACTAGAGTGTGA
GAGAGGGTTGTGGAATTCCCAGTGTAGAGGTGAAATTCGTAGATATTGGGAAGAACACCG
GTGGCGAAGGCGGCAACCTGGCTCATTACTGACGCTGAGGCGCGAAAGCGTGGGGAGC
>Acetobacter-2_TGTGCAG*
GGGCTAGCGTTGCTCGGAATGACTGGGCGTAAAGGGCGTGTAGGCGGTTTGTACAGTCAG
ATGTGAAATCCCCGGGCTTAACCTGGGAGCTGCATTTGATACGTGCAGACTAGAGTGTGA
GAGAGGGTTGTGGAATTCCCAGTGTAGAGGTGAAATTCGTAGATATTGGGAAGAACACCG
GTGGCGAAGGCGGCAACCTGGCTCATGACTGACGCTGAGGCGCGAAAGCGTGGGGAGC
>Acetobacter-3_TACGTAT*
GGGCTAGCGTTGCTCGGAATGACTGGGCGTAAAGGGCGTGTAGGCGGTTTACACAGTCAG
ATGTGAAATCCCCGGGCTTAACCTGGGAGCTGCATTTGATACGTGTAGACTAGAGTGTGA
GAGAGGGTTGTGGAATTCCCAGTGTAGAGGTGAAATTCGTAGATATTGGGAAGAACACCG
GTGGCGAAGGCGGCAACCTGGCTCATTACTGACGCTGAGGCGCGAAAGCGTGGGGAGC
>Acetobacter-4_TACGCAT*
GGGCTAGCGTTGCTCGGAATGACTGGGCGTAAAGGGCGTGTAGGCGGTTTACACAGTCAG
ATGTGAAATCCCCGGGCTTAACCTGGGAGCTGCATTTGATACGTGCAGACTAGAGTGTGA
GAGAGGGTTGTGGAATTCCCAGTGTAGAGGTGAAATTCGTAGATATTGGGAAGAACACCG
GTGGCGAAGGCGGCAACCTGGCTCATTACTGACGCTGAGGCGCGAAAGCGTGGGGAGC
>Acetobacter-5_TGTGCAA*
GGGCTAGCGTTGCTCGGAATGACTGGGCGTAAAGGGCGTGTAGGCGGTTTGTACAGTCAG
ATGTGAAATCCCCGGGCTTAACCTGGGAGCTGCATTTGATACGTGCAGACTAGAGTGTGA
GAGAGGGTTGTGGAATTCCCAGTGTAGAGGTGAAATTCGTAGATATTGGGAAGAACACCG
GTGGCGAAGGCGGCAACCTGGCTCATAACTGACGCTGAGGCGCGAAAGCGTGGGGAGC
>Acetobacter-6_TGTGGCG*
GGGCTAGCGTTGCTCGGAATGACTGGGCGTAAAGGGCGTGTAGGCGGTTTGTACAGTCAG
ATGTGAAATCCCCGGGCTTAACCTGGGAGCTGCATTTGATACGTGGCGACTAGAGTGTGA
GAGAGGGTTGTGGAATTCCCAGTGTAGAGGTGAAATTCGTAGATATTGGGAAGAACACCG
GTGGCGAAGGCGGCAACCTGGCTCATGACTGACGCTGAGGCGCGAAAGCGTGGGGAGC
>Acetobacter-7_TTGTAAT*
GGGCTAGCGTTGCTCGGAATGACTGGGCGTAAAGGGCGTGTAGGCGGTTTTGACAGTCAG
ATGTGAAATCCCCGGGCTTAACCTGGGAGCTGCATTTGAGACGTTAAGACTAGAGTGTGA
GAGAGGGTTGTGGAATTCCCAGTGTAGAGGTGAAATTCGTAGATATTGGGAAGAACACCG
GTGGCGAAGGCGGCAACCTGGCTCATTACTGACGCTGAGGCGCGAAAGCGTGGGGAGC
>isolate_209*
TGGCAAGCGTTATCCGGATTTATTGGGCGTAAAGCGAGCGCAGGCGGTTTTTTAAGTCTG
ATGTGAAAGCCCTCGGCTTAACCGAGGAAGTGCATCGGAAACTGGGAAACTTGAGTGCAG
AAGAGGACAGTGGAACTCCATGTGTAGCGGTGAAATGCGTAGATATATGGAAGAACACCA
GTGGCGAAGGCGGCTGTCTGGTCTGTAACTGACGCTGAGGCTCGAAAGCATGGGTAGC
>isolate_227
TGGCAAGCGTTATCCGGATTTATTGGGCGTAAAGCGAGCGCAGGCGGTCTTTTAAGTCTA
ATGTGAAAGCCTTCGGCTCAACCGAAGAAGTGCATTGGAAACTGGGAGACTTGAGTGCAG
AAGAGGACAGTGGAACTCCATGTGTAGCGGTGAAATGCGTAGATATATGGAAGAACACCA
GTGGCGAAGGCGGCTGTCTGGTCTGTAACTGACGCTAAGGCTCGAAAGCATGGGTAGC
>isolate_287

GGGCTAGCGTTGCTCGGAATGACTGGGCGTAAAGGGCGCGTAGGCGGTTGATGCAGTCAG
ATGTGAAATCCCCGGGCTTAACCTGGGAACTGCATTTGAGACGCATTGACTAGAGTTCGA
GAGAGGGTTGTGGAATTCCCAGTGTAGAGGTGAAATTCGTAGATATTGGGAAGAACACCG
GTGGCGAAGGCGGCAACCTGGCTCGATACTGACGCTGAGGCGCGAAAGCGTGGGGAGC
>isolate_299*
GTGCAAGCGTTAATCGGAATTACTGGGCGTAAAGCGCACGCAGGCGGTCTGTCAAGTCAG
ATGTGAAATCCCCGGGCTCAACCTGGGAACTGCATTCGAAACTGGCAGGCTAGAGTCTTG
TAGAGGGGGGTAGAATTCCAGGTGTAGCGGTGAAATGCGTAGAGATCTGGAGGAATACCG
GTGGCGAAGGCGGCCCCCTGGACAAAGACTGACGCTCATGTGCGAAAGCGTGGGGAGC
>isolate_301
GTGCACGCGTTACTCAGAATTACTGGGCGTAACGCGCACGCACGCTGTCTGTCATGTCAT
ATGTGATATCCCCGGTCTCAATGTGAGAACTGCATTCAAAACTGGCACGCTAGACTCTTG
TAGAGGGGGGTAGAATTCCGTGTGTAGCGGTGATGTGCATAGATATCTGGAAGAATACCG
GTGGCAAACGCCGCCCCCTGGACAGACACTGACTCTCATGTGCGAACGCGTGGAGAGC
>isolate_302
GTGCACGCGTTACTCAAAATTACTGGGCGTAACGCGCACGCACGCTGTCTGTCATCTCAG
ATGTGATCTCCCCGGGCTCAACCTGAGAACTGCATTCAAATCTGGCGCGCTAGACTCTTG
TAGAGGGGGGTAAAATTCCGTGTGTAGCTGTGATGTGCATAGATATCTAGAAGAATACCT
GTCGCAAACGCCGCCCCCTGGACAACACAGACTCTCATGTGCGACGCGTGGAGAGC
>isolate_307
GGGCTAGCGTTGCTCGGAATGACTGGGCGTAAAGGGCGTGTAGGCGGTTTGTACAGTCAG
ATGTGAAATCCCCGGGCTTAACCTGGGAGCTGCATTTGATACGTGCAGACTAGAGTGTGA
GAGAGGGTTGTGGAATTCCCAGTGTAGAGGTGAAATTCGTAGATATTGGGAAGAACACCG
GTGGCGAAGGCGGCAACCTGGCTCATGACTGACGCTGAGGCGCGAAAGCGTGGGGAGC
>isolate_308
TGGCAAGCGTTGTCCGGATTTATTGGGCGTAAAGCGAGCGCAGGCGGTTTTTTAAGTCTG
ATGTGAAAGCCTTCGGCTCAACCGAAGAAGTGCATCGGAAACTGGGAAACTTGAGTGCAG
AAGAGGACAGTGGAACTCCATGTGTAGCGGTGAAATGCGTAGATATATGGAAGAACACCA
GTGGCGAAGGCGGCTGTCTGGTCTGTAACTGACGCTGAGGCTCGAAAGTATGGGTAGC
>isolate_309
TGGCAAGCGTTGTCCGGATTTATTGGGCGTAAAGCGAGCGCAGGCGGTTTTTTAAGTCTG
ATGTGAAAGCCTTCGGCTCAACCGAAGAAGTGCATCGGAAACTGGGAAACTTGAGTGCAG
AAGAGGACAGTGGAACTCCATGTGTAGCGGTGAAATGCGTAGATATATGGAAGAACACCA
GTGGCGAAGGCGGCTGTCTGGTCTGTAACTGACGCTGAGGCTCGAAAGTATGGGTAGC
>isolate_310
GGGCTAGCGTTGCTCGGAATGACTGGGCGTAAAGGGCGTGTAGGCGGTTTGTACAGTCAG
ATGTGAAATCCCCGGGCTTAACCTGGGAGCTGCATTTGATACGTGCAGACTAGAGTGTGA
GAGAGGGTTGTGGAATTCCCAGTGTAGAGGTGAAATTCGTAGATATTGGGAAGAACACCG
GTGGCGAAGGCGGCAACCTGGCTCATGACTGACGCTGAGGCGCGAAAGCGTGGGGAGC
>isolate_311*
GGGCTAGCGTTGCTCGGAATGACTGGGCGTAAAGGGCGTGTAGGCGGTTTGTACAGTCAG
ATGTGAAATCCCCGGGCTTAACCTGGGAGCTGCATTTGATACGTGCAGACTAGAGTGTGA
GAGAGGGTTGTGGAATTCCCAGTGTAGAGGTGAAATTCGTAGATATTGGGAAGAACACCG
GTGGCGAAGGCGGCAACCTGGCTCATTACTGACGCTGAGGCGCGAAAGCGTGGGGAGC
>isolate_312
GGGCTAGCGTTGCTCGGAATGACTGGGCGTAAAGGGCGTGTAGGCGGTTTGTACAGTCAG
ATGTGAAATCCCCGGGCTTAACCTGGGAGCTGCATTTGATACGTGCAGACTAGAGTGTGA
GAGAGGGTTGTGGAATTCCCAGTGTAGAGGTGAAATTCGTAGATATTGGGAAGAACACCG
GTGGCGAAGGCGGCAACCTGGCTCATGACTGACGCTGAGGCGCGAAAGCGTGGGGAGC
>isolate_313*
GGGCTAGCGTTGCTCGGAATGACTGGGCGTAAAGGGCGTGTAGGCGGTTTGTACAGTCAG
ATGTGAAATCCCCGGGCTTAACCTGGGAGCTGCATTTGATACGTGCAGACTAGAGTGTGA
GAGAGGGTTGTGGAATTCCCAGTGTAGAGGTGAAATTCGTAGATATTGGGAAGAACACCG
GTGGCGAAGGCGGCAACCTGGCTCATGACTGACGCTGAGGCGCGAAAGCGTGGGGAGC

>isolate_314
TGGCAAGCGTTGTCCGGATTTATTGGGCGTAAAGCGAGCGCAGGCGGTTTTTTAAGTCTG
ATGTGAAAGCCTTCGGCTCAACCGAAGAAGTGCATCGGAAACTGGGAAACTTGAGTGCAG
AAGAGGACAGTGGAACTCCATGTGTAGCGGTGAAATGCGTAGATATATGGAAGAACACCA
GTGGCGAAGGCGGCTGTCTGGTCTGTAACTGACGCTGAGGCTCGAAAGTATGGGTAGC
>isolate_315
TGGCAAGCGTTGTCCGGATTTATTGGGCGTAAAGCGAGCGCAGGCGGTTTTTTAAGTCTG
ATGTGAAAGCCTTCGGCTCAACCGAAGAAGTGCATCGGAAACTGGGAAACTTGAGTGCAG
AAGAGGACAGTGGAACTCCATGTGTAGCGGTGAAATGCGTAGATATATGGAAGAACACCA
GTGGCGAAGGCGGCTGTCTGGTCTGTAACTGACGCTGAGGCTCGAAAGTATGGGTAGC
>isolate_318
TGGCAAGCGTTGTCCGGATTTATTGGGCGTAAAGCGAGCGCAGGCGGTTTCTTAAGTCTG
ATGTGAAAGCCCCCGGCTCAACCGGGGAGGGTCATTGGAAACTGGGAGACTTGAGTGCAG
AAGAGGAGAGTGGAATTCCATGTGTAGCGGTGAAATGCGTAGATATATGGAGGAACACCA
GTGGCGAAAGCGGCTCTCTGGTCTGTAACTGACGCTGAGGCTCGAAAGCGTGGGGAGC
>isolate_319
TGGCAAGCGTTATCCGGAATTATTGGGCGTAAAGCGCGCGCAGGCGGTTTCTTAAGTCTG
ATGTGAAAGCCCACGGCTCAACCGTGGAGGGTCATTGGAAACTGGGGAACTTGAGTGCAG
AAGAGAAAAGCGGAATTCCACGTGTAGCGGTGAAATGCGTAGAGATGTGGAGGAACACCA
GTGGCGAAGGCGGCTTTTTGGTCTGTAACTGACGCTGAGGCGCGAAAGCGTGGGGAGC
>isolate_321
TGGCAAGCGTTGTCCGGAATTATTGGGCGTAAAGGGCTCGCAGGCGGTTTCTTAAGTCTG
ATGTGAAAGCCCCCGGCTCAACCGGGGAGGGTCATTGGAAACTGGGGAACTTGAGTGCAG
AAGAGGAGAGTGGAATTCCACGTGTAGCGGTGAAATGCGTAGAGATGTGGAGGAACACCA
GTGGCGAAGGCGACTCTCTGGTCTGTAACTGACGCTGAGGAGCGAAAGCGTGGGGAGC
>isolate_46*
TGGCAAGCGTTGTCCGGATTTATTGGGCGTAAAGCGAGCGCAGGCGGTTTTTTAGGTCTG
ATGTGAAAGCCTTCGGCTTAACCGGAGAAGTGCATCGGAAACCGGGAAACTTGAGTGCAG
AAGAGGACAGTGGAACTCCATGTGTAGCGGTGAAATGCGTAGATATATGGAAGAACACCA
GTGGCGAAGGCGGCTGTCTGGTCTGCAACTGACGCTGAGGCTCGAAAGCATGGGTAGC
>isolate_56
TGGCAAGCGTTGTCCGGATTTATTGGGCGTAAAGCGAGCGCAGGCGGTTTTTTAAGTCTG
ATGTGAAAGCCTTCGGCTCAACCGAAGAAGTGCATCGGAAACTGGGAAACTTGAGTGCAG
AAGAGGACAGTGGAACTCCATGTGTAGCGGTGAAATGCGTAGATATATGGAAGAACACCA
GTGGCGAAGGCGGCTGTCTGGTCTGTAACTGACGCTGAGGCTCGAAAGTATGGGTAGC
>isolate_6
TGGCAAGCGTTATCCGGATTTATTGGGCGTAAAGCGAGCGCAGGCGGTCTTTTAAGTCTA
ATGTGAAAGCCTTCGGCTCAACCGAAGAAGTGCATTGGAAACTGGGAGACTTGAGTGCAG
AAGAGGACAGTGGAACTCCATGTGTAGCGGTGAAATGCGTAGATATATGGAAGAACACCA
GTGGCGAAGGCGGCTGTCTGGTCTGTAACTGACGCTGAGGCTCGAAAGCATGGGTAGC
>isolate_7
TGGCAAGCGTTATCCGGATTTATTGGGCGTAAAGCGAGCGCAGGCGGTCTTTTAAGTCTA
ATGTGAAAGCCTTCGGCTCAACCGAAGAAGTGCATTGGAAACTGGGAGACTTGAGTGCAG
AAAAGGACAGTGGAACTCCATGTGTAGCGGTGAAAGCGTAGATATATGGAAGAACACCAG
TGGCGAAGCGGCTGTCGGTCTGAACTGACGCTGAGGCTCGAAGCTGGGTAGCGAACAGGA
>isolate_286
GGGCTAGCGTTGCTCGGAATGACTGGGCGTAAAGGGCGCGTAGGCGGTTGATGCAGTCAG
ATGTGAAATCCCCGGGCTTAACCTGGGAACTGCATTTGAGACGCATTGACTAGAGTTCGA
GAGAGGGTTGTGGAATTCCCAGTGTAGAGGTGAAATTCGTAGATGTTGGGAAGAACACCG
GTGGCGAAGGCGGCAACCTGGCTCGATACTGACGCTGAGGCGCGAAAGCGTGGGGAGC
>isolate_13
TGGCAAGCGTTATCCGGATTTATTGGGCGTAAAGCGAGCGCAGGCGGTCTTTTAAGTCTA
ATGTGAAAGCCTTCGGCTCAACCGAAGAAGTGCATTGGAAACTGGGAGACTTGAGTGCAG
AAGAGGACAGTGGAACTCCATGTGTAGCGGTGAAATGCGTAGATATATGGAAGAACACCA

GTGGCGAAGGCGGCTGTCTGGTCTGTAACTGACGCTGAGGCTCGAAAGCATGGGTAGC
>isolate_24*
TGGCCAGCGTTGTCCGGATTTATTGGGCGTAAAGCGAGCGCAGGCGGTTTTTTAAGTCTG
ATGTGAAAGCCTTCGGCTTAACCGGAGAAGTGCATCGGAAACTGGGAGACTTGAGTGCAG
AAGAGGACAGTGGAACTCCATGTGTAGCGGTGGAATGCGTAGATATATGGAAGAACACCA
GTGGCGAAGGCGGCTGTCTAGTCTGTAACTGACGCTGAGGCTCGAAAGCATGGGTAGC
>isolate_34*
TCCCGAGCGTTATCCGGATTTATTGGGCGTAAAGCGAGCGCAGACGGTTTATTAAGTCTG
ATGTGAAAGCCCGGAGCTCAACTCCGGAATGGCATTGGAAACTGGTTAACTTGAGTGCAG
TAGAGGTAAGTGGAACTCCATGTGTAGCGGTGGAATGCGTAGATATATGGAAGAACACCA
GTGGCGAAGGCGGCTTACTGGACTGCAACTGACGTTGAGGCTCGAAAGTGTGGGTAGC
>isolate_44
TGGCAAGCGTTATCCGGATTTATTGGGCGTAAAGCGAGCGCAGGCGGTCTTTTAAGTCTA
ATGTGAAAGCCTTCGGCTCAACCGAAGAAGTGCATTGGAAACTGGGAGACTTGAGTGCAG
AAGAGGACAGTGGAACTCCATGTGTAGCGGTGAAATGCGTAGATATATGGAAGAACACCA
GTGGCGAAGGCGGCTGTCTGGTCTGTAACTGACGCTGAGGCTCGAAAGCATGGGTAGC
>isolate_12
TGGCAAGCGTTATCCGGATTTATTGGGCGTAAAGCGAGCGCAGGCGGTCTTTTAAGTCTA
ATGTGAAAGCCTTCGGCTCAACCGAAGAAGTGCATTGGAAACTGGGAGACTTGAGTGCAG
AAGAGGACAGTGGAACTCCATGTGT
>isolate_300*
GTGCAAGCGTTAATCGGAATTACTGGGCGTAAAGCGCACGCAGGCGGTCTGTCAAGTCGG
ATGTGAAATCCCCGGGCTCAACCCGGGAACTGCATTCGAAACTGGCAGGCTAGAGTCTTG
TAGAGGGGGGTAGAATTCCAGGTGTAGCGGTGAAATGCGTAGAGATCTGGAGGAATACC
GGTGGCGAAGGCGGCCCCCTGGACAAAGACTGACGCTCAGGTGCGAAAGCGTGGGGAGC
>isolate_316
TGGCAAGCGTTGTCCGGAATTATTGGGCGTAAAGCGCGCGCAGGCGGCTTCTTAAGTCTG
ATGTGAAATCTTGCGGCTCAACCGCAAGCGGTCATTGGAAACTGGGAGGCTTGAGTGCAG
AAGAGGAGAGTGGAATTCCACGTGTAGCGGTGAAATGCGTAGAGATGTGGAGGAACACCA
GTGGCGAAAGGCGGCTCTCTGGTCTGTAACTGACGCTGA
>isolate_322
TGGCAAGCGTTGTCCGGAATTATTGGGCGTAAAGGGCTCGCAGGCGGTTTCTTAAGTCTG
ATGTGAAAGCCCCCGGCTCAACCGGGGAGGGTCATTGGAAACTGGGGAACTTGAGTGCAG
AAGAGGAGAGTGGAATTCCACGTGTAGCGGTGAAATGCGTAGAGATGTGGAGGAACACCA
GTGGCGAAAGGCGACTCTCTGGTCTGTAACTGACGCTGAGGAGCGAAAGCGTGGGGAGC
>isolate_1*
TGGCAAGCGTTATCCGGATTTATTGGGCGTAAAGAGAGTGCAGGCGGTTTTCTAAGTCTG
ATGTGAAAGCCTTCGGCTTAACCGGAGAAGTGCATCGGAAACTGGATAACTTGAGTGCAG
AAGAGGGTAGTGGAACTCCATGTGTAGCGGTGGAATGCGTAGATATATGGAAGAACACCA
GTGGCGAAGGCGGCTACCTGGTCTGCAACTGACGCTGAGACTCGAAAGCATGGGTAGC
>isolate_2
TGGCAAGCGTTGTCCGGATTTATTGGGCGTAAAGCGAGCGCAGGCGGTTTTTTAAGTCTG
ATGTGAAAGCCTTCGGCTCAACCGAAGAAGTGCATCGGAAACTGGGAAACTTGAGTGCAG
AAGAGGACAGTGGAACTCCATGTGTAGCGGTGAAATGCGTAGATATATGGAAGAACACCA
GTGGCGAAGGCGGCTGTCTGGTCTGTAACTGACGCTGAGGCTCGAAAGTATGGGTAGC
>isolate_3
TGGCAAGCGTTATCCGGATTTATTGGGCGTAAAGAGAGTGCAGGCGGTTTTCTAAGTCTG
ATGTGAAAGCCTTCGGCTTAACCGGAGAAGTGCATCGGAAACTGGATAACTTGAGTGCAG
AAGAGGGTAGTGGAACTCCATGTGTAGCGGTGGAATGCGTAGATATATGGAAGAACACCA
GTGGCGAAGGCGGCTACCTGGTCTGCAACTGACGCTGAGACTCGAAAGCATGGGTAGC
>isolate_4
TGGCAAGCGTTATCCGGATTTATTGGGCGTAAAGAGAGTGCAGGCGGTTTTCTAAGTCTG
ATGTGAAAGCCTTCGGCTTAACCGGAGAAGTGCATCGGAAACTGGATAACTTGAGTGCAG
AAGAGGGTAGTGGAACTCCATGTGTAGCGGTGGAATGCGTAGATATATGGAAGAACACCA

GTGGCGAAGGCGGCTACCTGGTCTGCAACTGACGCTGAGACTCGAAAGCATGGGTAGC
>isolate_5*
TGGCAAGCGTTATCCGGATTTATTGGGCGTAAAGAGAGTGCAGGCGGTTTTCTAAGTCTG
ATGTGAAAGCCTTCGGCTTAACCGGAGAAGTGCATCGGAAACTGGATAACTTGAGTGCAG
AAGAGGGTAGTGGAACTCCATGTGTAGCGGTGGAATGCGTAGATATATGGAAGAACACCA
GTGGCGAAGGCGGCTACCTGGTCTGCAACTGACGCTGAGACTCGAAAGCATGGGTAGC
>isolate_6
TGGCAAGCGTTATCCGGATTTATTGGGCGTAAAGAGAGTGCAGGCGGTTTTCTAAGTCTG
ATGTGAAAGCCTTCGGCTTAACCGGAGAAGTGCATCGGAAACTGGATAACTTGAGTGCAG
AAGAGGGTAGTGGAACTCCATGTGTAGCGGTGGAATGCGTAGATATATGGAAGAACACCA
GTGGCGAAGGCGGCTACCTGGTCTGCAACTGACGCTGAGACTCGAAAGCATGGGTAGC
>isolate_7
TGGCAAGCGTTATCCGGATTTATTGGGCGTAAAGAGAGTGCAGGCGGTTTTCTAAGTCTG
ATGTGAAAGCCTTCGGCTTAACCGGAGAAGTGCATCGGAAACTGGATAACTTGAGTGCAG
AAGAGGGTAGTGGAACTCCATGTGTAGCGGTGGAATGCGTAGATATATGGAAGAACACCA
GTGGCGAAGGCGGCTACCTGGTCTGCAACTGACGCTGAGACTCGAAAGCATGGGTAGC
>isolate_8
TGGCAAGCGTTATCCGGATTTATTGGGCGTAAAGAGAGTGCAGGCGGTTTTCTAAGTCTG
ATGTGAAAGCCTTCGGCTTAACCGGAGAAGTGCATCGGAAACTGGATAACTTGAGTGCAG
AAGAGGGTAGTGGAACTCCATGTGTAGCGGTGGAATGCGTAGATATATGGAAGAACACCA
GTGGCGAAGGCGGCTACCTGGTCTGCAACTGACGCTGAGACTCGAAAGCATGGGTAGC
>isolate_9
TGGCAAGCGTTATCCGGATTTATTGGGCGTAAAGAGAGTGCAGGCGGTTTTCTAAGTCTG
ATGTGAAAGCCTTCGGCTTAACCGGAGAAGTGCATCGGAAACTGGATAACTTGAGTGCAG
AAGAGGGTAGTGGAACTCCATGTGTAGCGGTGGAATGCGTAGATATATGGAAGAACACCA
GTGGCGAAGGCGGCTACCTGGTCTGCAACTGACGCTGAGACTCGAAAGCATGGGTAGC
>isolate_10
TGGCAAGCGTTATCCGGATTTATTGGGCGTAAAGAGAGTGCAGGCGGTTTTCTAAGTCTG
ATGTGAAAGCCTTCGGCTTAACCGGAGAAGTGCATCGGAAACTGGATAACTTGAGTGCAG
AAGAGGGTAGTGGAACTCCATGTGTAGCGGTGGAATGCGTAGATATATGGAAGAACACCA
GTGGCGAAGGCGGCTACCTGGTCTGCAACTGACGCTGAGACTCGAAAGCATGGGTAGC
>isolate_11
TGGCAAGCGTTATCCGGATTTATTGGGCGTAAAGAGAGTGCAGGCGGTTTTCTAAGTCTG
ATGTGAAAGCCTTCGGCTTAACCGGAGAAGTGCATCGGAAACTGGATAACTTGAGTGCAG
AAGAGGGTAGTGGAACTCCATGTGTAGCGGTGGAATGCGTAGATATATGGAAGAACACCA
GTGGCGAAGGCGGCTACCTGGTCTGCAACTGACGCTGAGACTCGAAAGCATGGGTAGC
>isolate_12
TGGCAAGCGTTATCCGGATTTATTGGGCGTAAAGAGAGTGCAGGCGGTTTTCTAAGTCTG
ATGTGAAAGCCTTCGGCTTAACCGGAGAAGTGCATCGGAAACTGGATAACTTGAGTGCAG
AAGAGGGTAGTGGAACTCCATGTGTAGCGGTGGAATGCGTAGATATATGGAAGAACACCA
GTGGCGAAGGCGGCTACCTGGTCTGCAACTGACGCTGAGACTCGAAAGCATGGGTAGC
>isolate_13
TGGCAAGCGTTATCCGGATTTATTGGGCGTAAAGAGAGTGCAGGCGGTTTTCTAAGTCTG
ATGTGAAAGCCTTCGGCTTAACCGGAGAAGTGCATCGGAAACTGGATAACTTGAGTGCAG
AAGAGGGTAGTGGAACTCCATGTGTAGCGGTGGAATGCGTAGATATATGGAAGAACACCA
GTGGCGAAGGCGGCTACCTGGTCTGCAACTGACGCTGAGACTCGAAAGCATGGGTAGC
>isolate_14
TGGCAAGCGTTATCCGGATTTATTGGGCGTAAAGAGAGTGCAGGCGGTTTTCTAAGTCTG
ATGTGAAAGCCTTCGGCTTAACCGGAGAAGTGCATCGGAAACTGGATAACTTGAGTGCAG
AAGAGGGTAGTGGAACTCCATGTGTAGCGGTGGAATGCGTAGATATATGGAAGAACACCA
GTGGCGAAGGCGGCTACCTGGTCTGCAACTGACGCTGAGACTCGAAAGCATGGGTAGC
>isolate_15
TGGCAAGCGTTATCCGGATTTATTGGGCGTAAAGAGAGTGCAGGCGGTTTTCTAAGTCTG
ATGTGAAAGCCTTCGGCTTAACCGGAGAAGTGCATCGGAAACTGGATAACTTGAGTGCAG

AAGAGGGTAGTGGAACTCCATGTGTAGCGGTGGAATGCGTAGATATATGGAAGAACACCA
GTGGCGAAGGCGGCTACCTGGTCTGCAACTGACGCTGAGACTCGAAAGCATGGGTAGC
>isolate_16
TGGCAAGCGTTATCCGGATTTATTGGGCGTAAAGAGAGTGCAGGCGGTTTTCTAAGTCTG
ATGTGAAAGCCTTCGGCTTAACCGGAGAAGTGCATCGGAAACTGGATAACTTGAGTGCAG
AAGAGGGTAGTGGAACTCCATGTGTAGCGGTGGAATGCGTAGATATATGGAAGAACACCA
GTGGCGAAGGCGGCTACCTGGTCTGCAACTGACGCTGAGACTCGAAAGCATGGGTAGC
>isolate_17
TGGCAAGCGTTATCCGGATTTATTGGGCGTAAAGAGAGTGCAGGCGGTTTTCTAAGTCTG
ATGTGAAAGCCTTCGGCTTAACCGGAGAAGTGCATCGGAAACTGGATAACTTGAGTGCAG
AAGAGGGTAGTGGAACTCCATGTGTAGCGGTGGAATGCGTAGATATATGGAAGAACACCA
GTGGCGAAGGCGGCTACCTGGTCTGCAACTGACGCTGAGACTCGAAAGCATGGGTAGC
>isolate_18*
TGGCAAGCGTTGTCCGGATTTATTGGGCGTAAAGCGAGCGCAGGCGGTTTTTTAAGTCTG
ATGTGAAAGCCTTCGGCTCAACCGAAGAAGTGCATCGGAAACTGGGAAACTTGAGTGCAG
AAGAGGACAGTGGAACTCCATGTGTAGCGGTGAAATGCGTAGATATATGGAAGAACACCA
GTGGCGAAGGCGGCTGTCTGGTCTGTAACTGACGCTGAGGCTCGAAAGTATGGGTAGC
>isolate_19*
TGGCAAGCGTTGTCCGGATTTATTGGGCGTAAAGGGAACGCAGGCGGTCTTTTAAGTCTG
ATGTGAAAGCCTTCGGCTTAACCGAAGTCGTGCATTGGAAACTGGGAGACTTGAGTGCAG
AAGAGGAGAGTGGAACTCCATGTGTAGCGGTGAAATGCGTAGATATATGGAAGAACACCA
GTGGCGAAAGCGGCTCTCTGGTCTGTAACTGACGCTGAGGTTCGAAAGCGTGGGTAGC
>isolate_28
GGGCTAGCGTTGCTCGGAATGACTGGGCGTAAAGGGCGTGTAGGCGGTTTGTACAGTCAG
ATGTGAAATCCCCGGGCTTAACCTGGGAGCTGCATTTGATACGTGCAGACTAGAGTGTGA
GAGAGGGTTGTGGAATTCCCAGTGTAGAGGTGAAATTCGTAGATATTGGGAAGAACACCG
GTGGCGAAGGCGGCAACCTGGCTCATTACTGACGCTGAGGCGCGAAAGCGTGGGGAGC
>isolate_29
GGGCTAGCGTTGCTCGGAATGACTGGGCGTAAAGGGCGTGTAGGCGGTTTGTACAGTCAG
ATGTGAAATCCCCGGGCTTAACCTGGGAGCTGCATTTGATACGTGCAGACTAGAGTGTGA
GAGAGGGTTGTGGAATTCCCAGTGTAGAGGTGAAATTCGTAGATATTGGGAAGAACACCG
GTGGCGAAGGCGGCAACCTGGCTCATTACTGACGCTGAGGCGCGAAAGCGTGGGGAGC
>isolate_35
GGGCTAGCGTTGCTCGGAATGACTGGGCGTAAAGGGCGTGTAGGCGGTTTGTACAGTCAG
ATGTGAAATCCCCGGGCTTAACCTGGGAGCTGCATTTGATACGTGCAGACTAGAGTGTGA
GAGAGGGTTGTGGAATTCCCAGTGTAGAGGTGAAATTCGTAGATATTGGGAAGAACACCG
GTGGCGAAGGCGGCAACCTGGCTCATTACTGACGCTGAGGCGCGAAAGCGTGGGGAGC
>isolate_36
GGGCTAGCGTTGCTCGGAATGACTGGGCGTAAAGGGCGTGTAGGCGGTTTGTACAGTCAG
ATGTGAAATCCCCGGGCTTAACCTGGGAGCTGCATTTGATACGTGCAGACTAGAGTGTGA
GAGAGGGTTGTGGAATTCCCAGTGTAGAGGTGAAATTCGTAGATATTGGGAAGAACACCG
GTGGCGAAGGCGGCAACCTGGCTCATTACTGACGCTGAGGCGCGAAAGCGTGGGGAGC
>isolate_37
TGGCAAGCGTTATCCGGATTTATTGGGCGTAAAGCGAGCGCAGGCGGTCTTTTAAGTCTA
ATGTGAAAGCCTTCGGCTCAACCGAAGAAGTGCATTGGAAACTGGGAGACTTGAGTGCAG
AAGAGGACAGTGGAACTCCATGTGTAGCGGTGAAATGCGTAGATATATGGAAGAACACCA
GTGGCGAAGGCGGCTGTCTGGTCTGTAACTGACGCTGAGGCTCGAAAGCATGGGTAGC
>isolate_40
GGGCTAGCGTTGCTCGGAATGACTGGGCGTAAAGGGCGTGTAGGCGGTTTGTACAGTCAG
ATGTGAAATCCCCGGGCTTAACCTGGGAGCTGCATTTGATACGTGCAGACTAGAGTGTGA
GAGAGGGTTGTGGAATTCCCAGTGTAGAGGTGAAATTCGTAGATATTGGGAAGAACACCG
GTGGCGAAGGCGGCAACCTGGCTCATTACTGACGCTGAGGCGCGAAAGCGTGGGGAGC
>isolate_41
GGGCTAGCGTTGCTCGGAATGACTGGGCGTAAAGGGCGTGTAGGCGGTTTGTACAGTCAG

ATGTGAAATCCCCGGGCTTAACCTGGGAGCTGCATTTGATACGTGCAGACTAGAGTGTGA
GAGAGGGTTGTGGAATTCCCAGTGTAGAGGTGAAATTCGTAGATATTGGGAAGAACACCG
GTGGCGAAGGCGGCAACCTGGCTCATTACTGACGCTGAGGCGCGAAAGCGTGGGGAGC
>isolate_43
GGGCTAGCGTTGCTCGGAATGACTGGGCGTAAAGGGCGTGTAGGCGGTTTGTACAGTCAG
ATGTGAAATCCCCGGGCTTAACCTGGGAGCTGCATTTGATACGTGCAGACTAGAGTGTGA
GAGAGGGTTGTGGAATTCCCAGTGTAGAGGTGAAATTCGTAGATATTGGGAAGAACACCG
GTGGCGAAGGCGGCAACCTGGCTCATTACTGACGCTGAGGCGCGAAAGCGTGGGGAGC
>isolate_45
GGGCTAGCGTTGCTCGGAATGACTGGGCGTAAAGGGCGTGTAGGCGGTTTGTACAGTCAG
ATGTGAAATCCCCGGGCTTAACCTGGGAGCTGCATTTGATACGTGCAGACTAGAGTGTGA
GAGAGGGTTGTGGAATTCCCAGTGTAGAGGTGAAATTCGTAGATATTGGGAAGAACACCG
GTGGCGAAGGCGGCAACCTGGCTCATTACTGACGCTGAGGCGCGAAAGCGTGGGGAGC
>isolate_49*
TGGCAAGCGTTGTCCGGATTTATTGGGCGTAAAGGGAACGCAGGCGGTTTTTTAAGTCTG
ATGTGAAAGCCTTCGGCTTAACCGAAGTCGTGCATTGGAAACTGGAGAACTTGAGTGCAG
AAGAGGAGAGTGGAACTCCATGTGTAGCGGTGAAATGCGTAGATATATGGAAGAACACCA
GTGGCGAAAGCGGCTCTCTGGTCTGTAACTGACGCTGAGGTTCGAAAGCGTGGGTAGC
>isolate_57
GGGCTAGCGTTGCTCGGAATGACTGGGCGTAAAGGGCGTGTAGGCGGTTTGTACAGTCAG
ATGTGAAATCCCCGGGCTTAACCTGGGAGCTGCATTTGATACGTGCAGACTAGAGTGTGA
GAGAGGGTTGTGGAATTCCCAGTGTAGAGGTGAAATTCGTAGATATTGGGAAGAACACCG
GTGGCGAAGGCGGCAACCTGGCTCATTACTGACGCTGAGGCGCGAAAGCGTGGGGAGC
>isolate_61
GGGCTAGCGTTGCTCGGAATGACTGGGCGTAAAGGGCGTGTAGGCGGTTTGTACAGTCAG
ATGTGAAATCCCCGGGCTTAACCTGGGAGCTGCATTTGATACGTGCAGACTAGAGTGTGA
GAGAGGGTTGTGGAATTCCCAGTGTAGAGGTGAAATTCGTAGATATTGGGAAGAACACCG
GTGGCGAAGGCGGCAACCTGGCTCATTACTGACGCTGAGGCGCGAAAGCGTGGGGAGC
