# Supplementary material for: Fine Resolution Analysis of Microbial Communities Provides Insights Into the Variability of Cocoa Bean Fermentation
Source: Front Microbiol. 2020 Apr 15;11:650. doi: 10.3389/fmicb.2020.00650 (PMC7174660; doi:10.3389/fmicb.2020.00650)
Supplement: Supplementary file 2 [file Data_Sheet_2.PDF]

Supplementary Material

1.1 Supplementary Figures

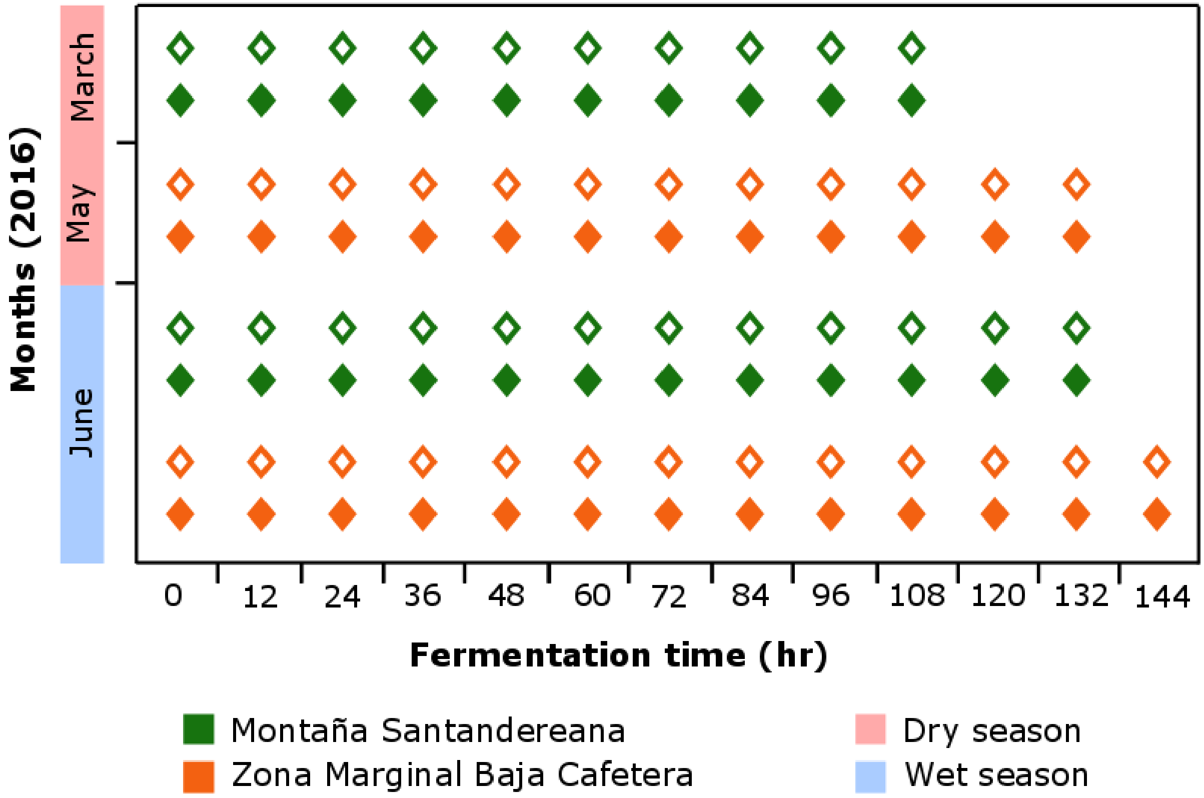

Figure S1. Fermentation processes of cocoa bean sampled in two model farms from two agroecological zones from Colombia. A simple schematic of the sampling times (in hours) is shown. Microbial monitoring of the cocoa bean was carried out in two agroecological zones from Colombia, *Montaña Santandereana* (MS), and *Zona Marginal Baja Cafetera* (BC), colored green and orange, respectively. The sampling was realized in three months (2016), dry season (pink) and wet season (blue), and beans were collected from upper (filled diamond) and middle (open diamond) sections of the wooden box every 12 hours from the start to the end of fermentation.

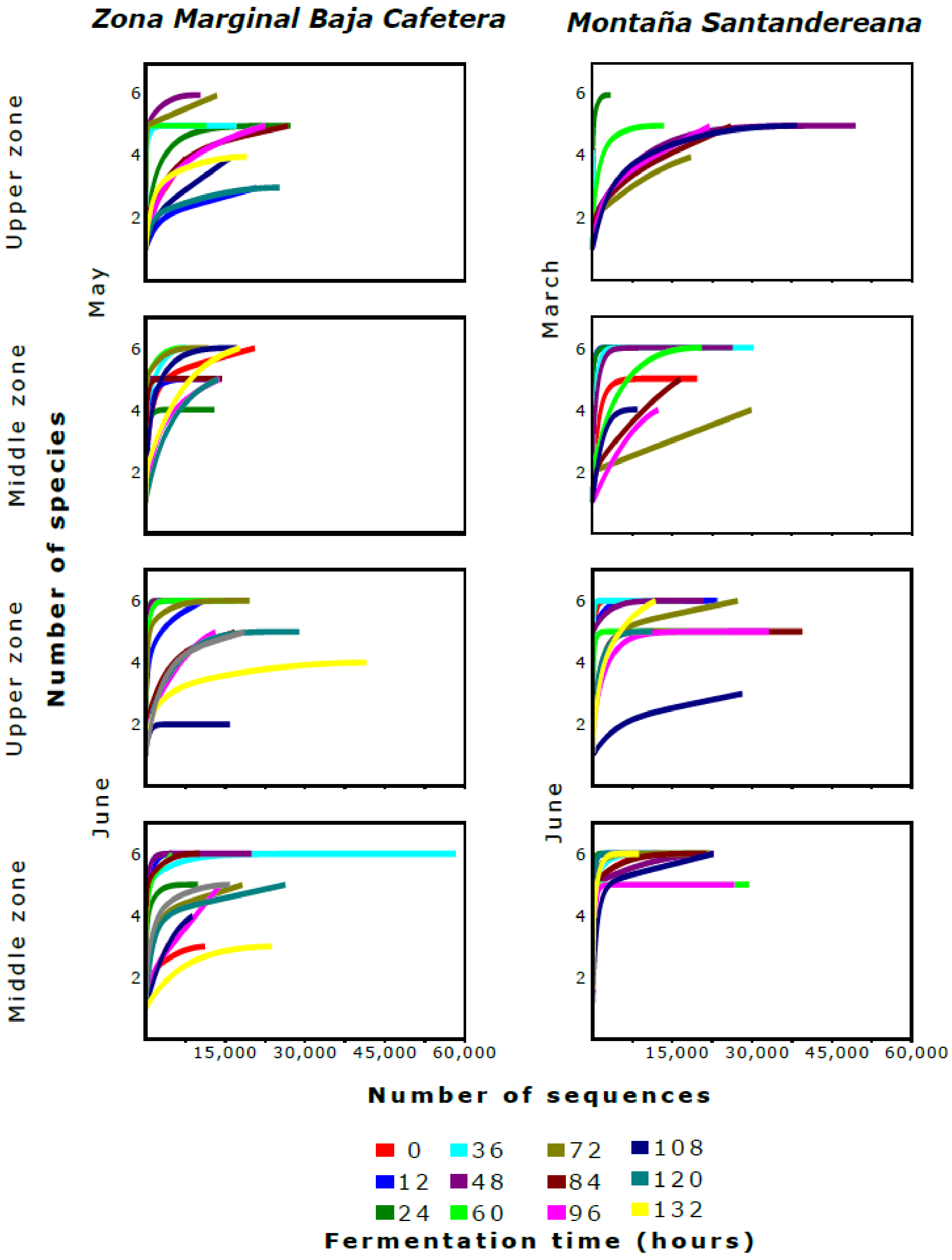

Figure S2. Rarefaction analysis of 16S rRNA gene libraries. Rarefaction curves for the cumulative observation of species on each sampling time for each fermentation process is shown for both AEZs. Even though the highest sequencing depth per sample varied between 45,000 and 60,000 reads, an average at of ~15,000 reads per sample are observed. Notice that even in low sequencing effort samples, a saturation is achieved due to the low number of OTUs present.

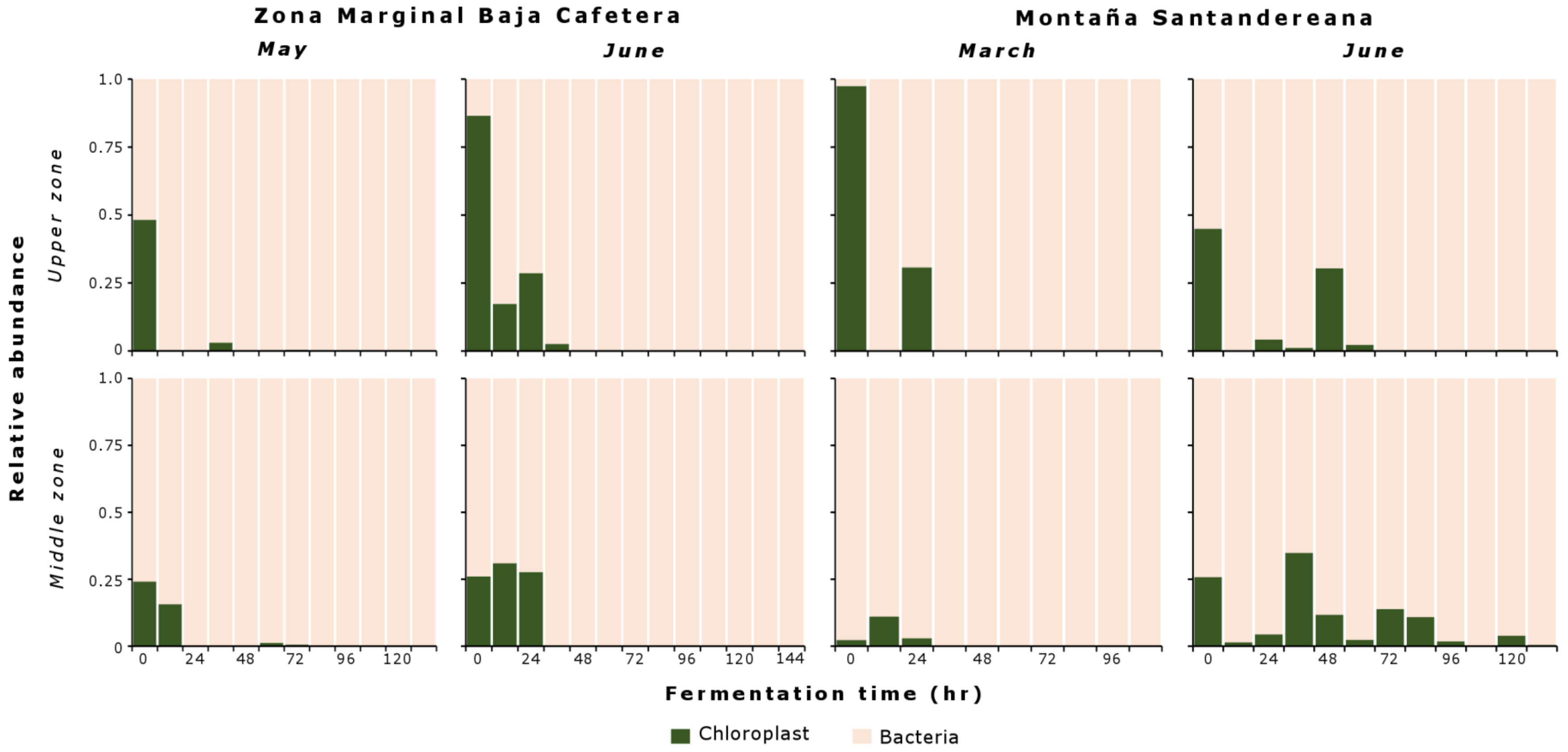

Figure S3. Variation of 16S rRNA gene reads proportion assigned to bacteria and chloroplast during fermentation time for each fermentation process. The proportion of 16S rRNA gene amplicon reads assigned to chloroplast reflects the plant transformation during the process. All chloroplast reads were assigned to *Theobroma cacao*.

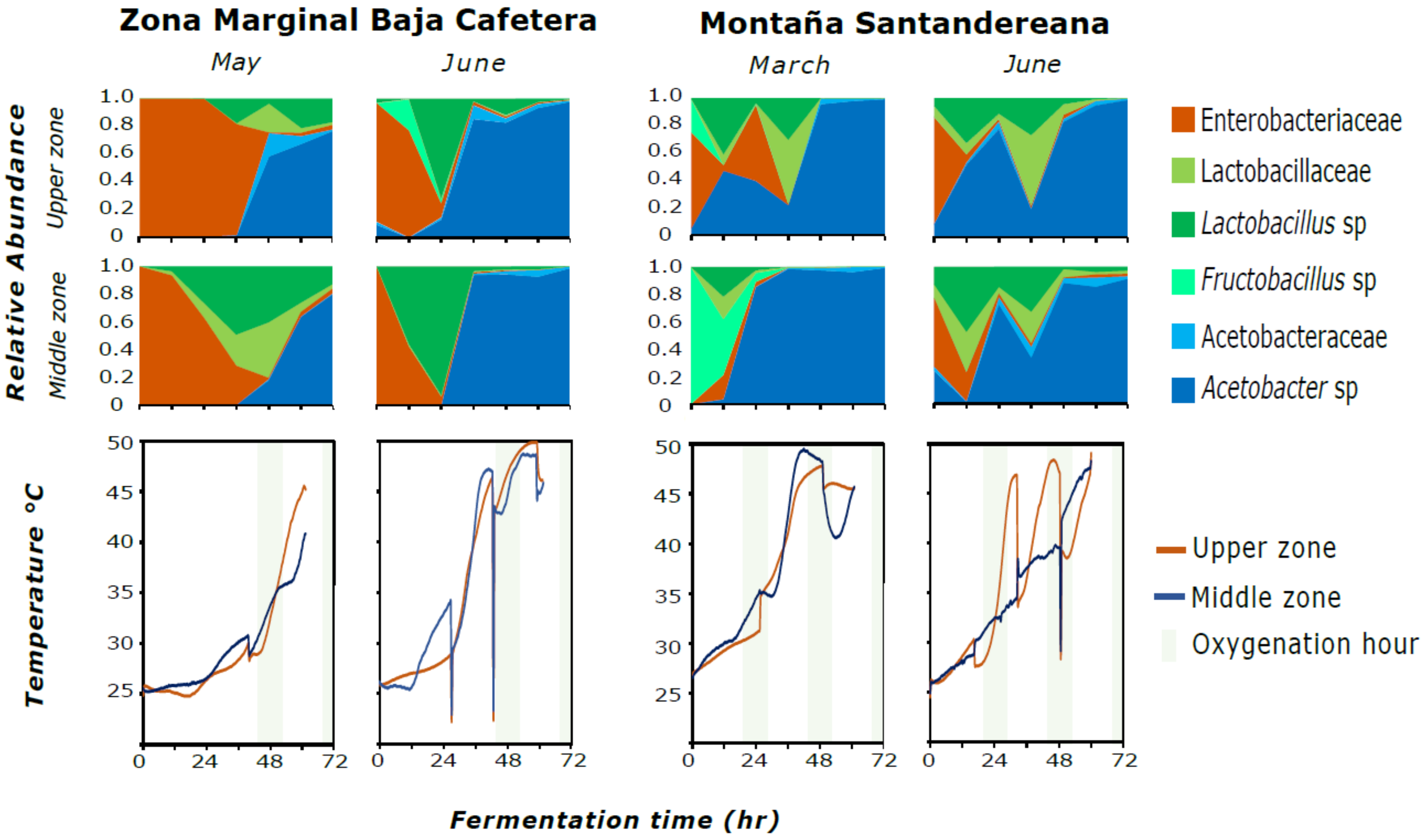

Figure S4. Impact of oxygen introduction by bean mixing in the microbial succession. Upper panels show the bacterial OTUs relative abundances for the different fermentation processes as shown in **Figure 1**. Matching bottom panels show for each process the variation in temperature for Upper and Middle zones as well as the time of oxygenation, by mixing, as shaded columns.

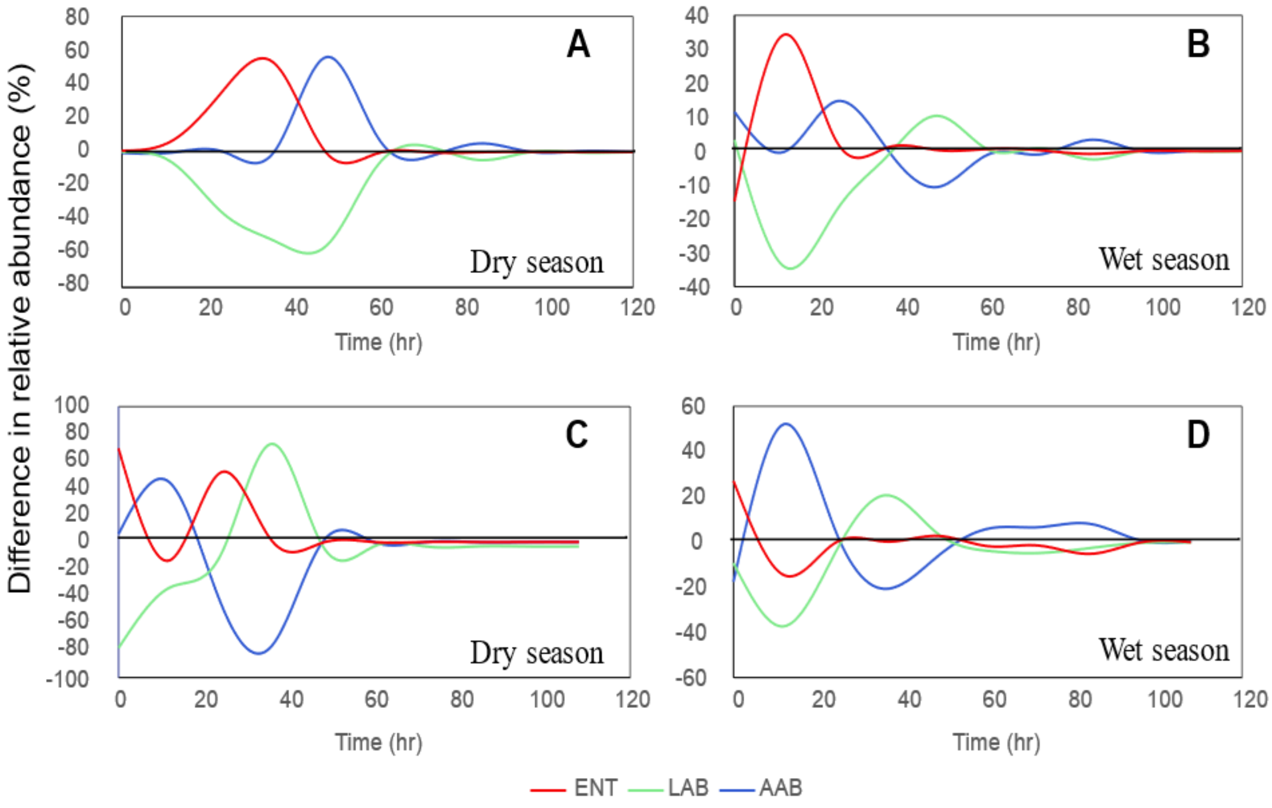

Figure S5. Difference in the relative abundance of bacterial groups from the upper and middle zone of cocoa bean mass during fermentation. The values are shown for BC fermentations during May (A) and June (B), and for MS during March (C) and June (D). The difference in the relative abundance of bacteria was determined for enterobacteria (ENT), lactic acid bacteria (LAB) and acetic acid bacteria (AAB), by subtracting the middle zone relative abundance from the upper zone, values lower than zero show higher values abundance in the middle zone, and higher than zero show higher relative abundance in the upper zone.

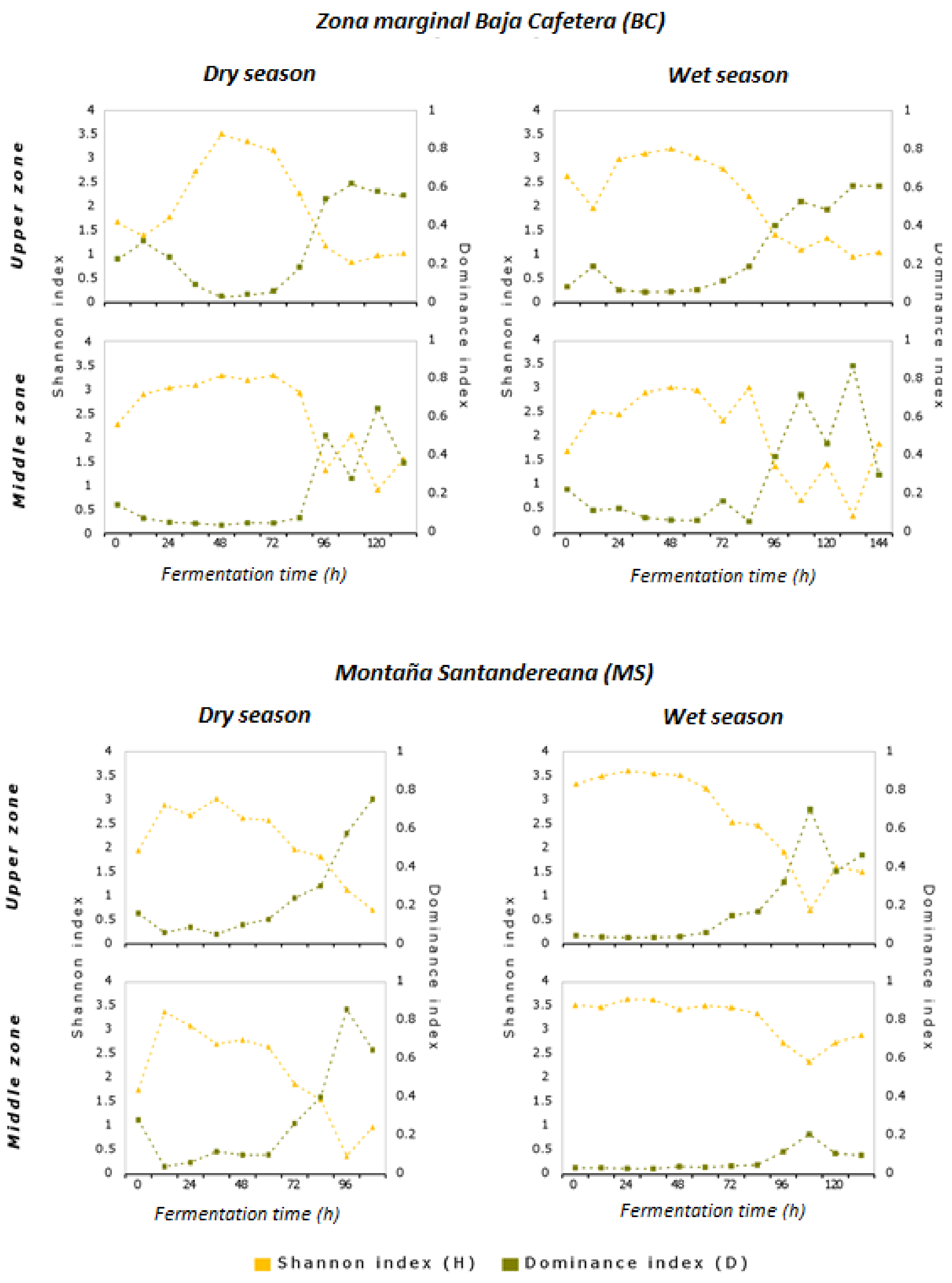

Figure S6. Changes of diversity and dominance of oligotypes for each fermentation process. For each fermentation process the fluctuation of the Shannon index and Dominance index was measured on all oligotypes and their abundances. Both diversity indexes were calculated using PAST v3.

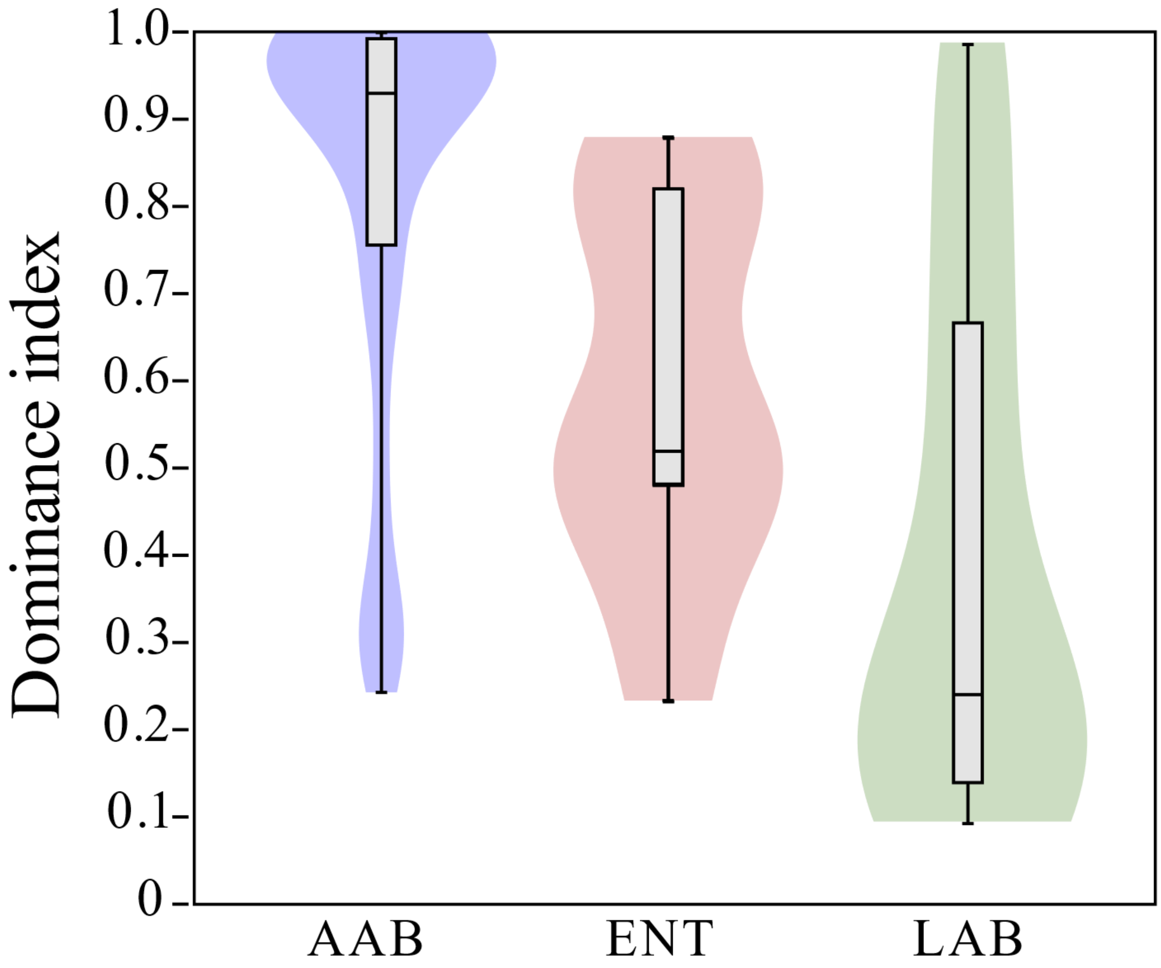

Figure S7. Distribution of dominance index within the different bacterial groups. The dominance index was quantified for samples where the relative abundance of the group was higher that 10%., The figure shows the distributions as a violin plot and a box plot for the Acetic Acid Bacteria (AAB), Enterobacteriaceae (ENT) and Lactic Acid Bacteria (LAB).

.

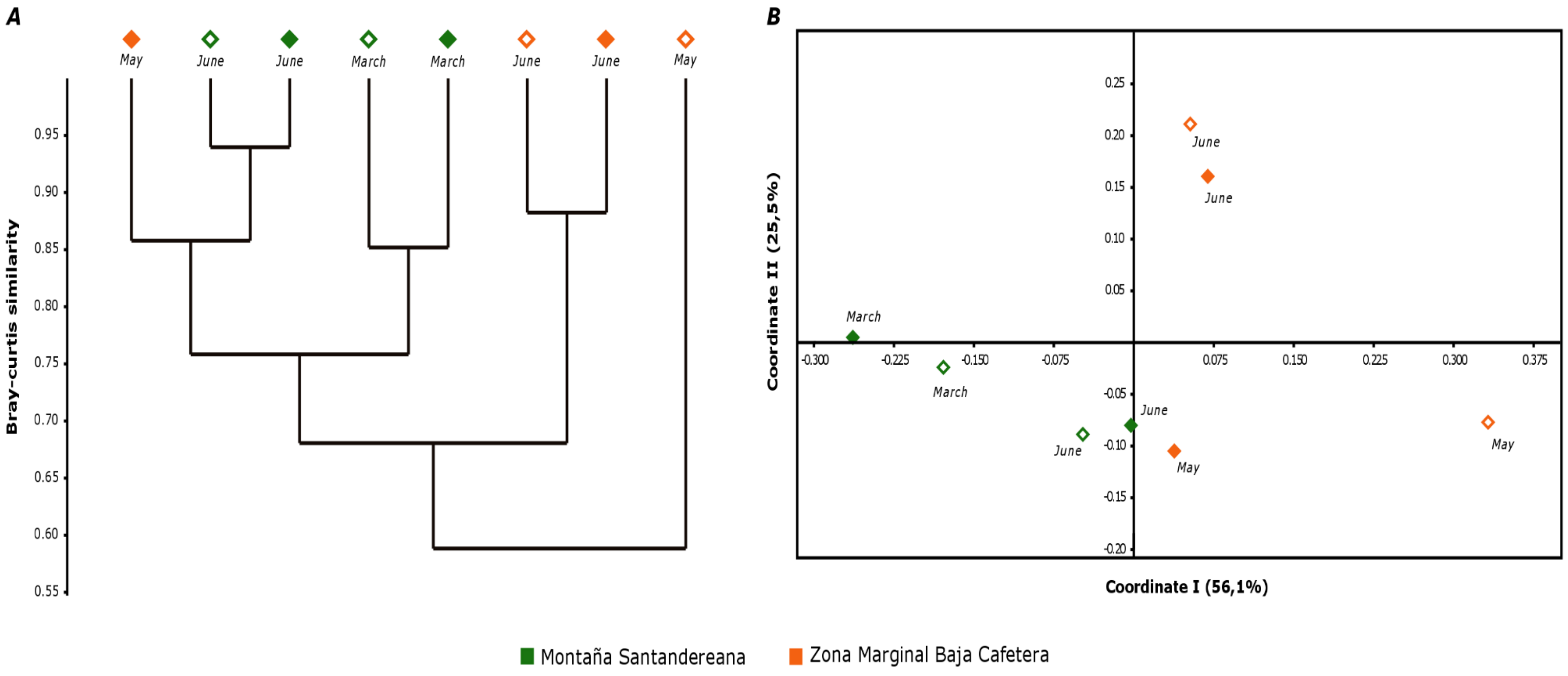

Figure S8. Multivariate analyses of the correlation between all fermentation processes. UPGMA clustering (A) and Principal Coordinates Analyses (B) based on the Bray-Curtis dissimilarity index of the oligotypes abundance from the oligotypes was calculated for all fermentation processes. Each fermentation process is identified by shapes and colors as in Fig. S 1. Both analyses were made in PAST

1.2 Supplementary Tables

Table S1. Features of agroecological zones used during the study.

Feature	Agroecological zone	
	**Montaña Santandereana**	**Zona Marginal Baja Cafetera**
**Location**	Santander, Norte de Santander	Caldas, Quindío, Risaralda, Antioquia, Cundinamarca, Santander, north of Tolima and Boyacá
**Altitude (m.a.s.l.)**	500 - 1000	900 - 1200
**Temperature (°C)**	23- 28	23 - 27
**Precipitation (mm/year)**	1500 - 2000	1800 - 2200
**Soil types**	Loose and slopes soils, with a medium to low fertility and a broken topography	Clayey or loamy-clayey, predominantly in hillside and deep
**Localization of model farm**	Department: Santander; town: San Vicente de Chucurí; farm: La Belleza	Department: Antioquia; town: Maceo, farm: Cannes
**Department harvest area (Ha)/cocoa production (Ton)**	45,472 / 23,430	15,491 / 9,020
**National production participation (%) of Department**	26.85	10.34
**Assessment of the cocoa bean quality after the fermentation process**		
**Fermented grains (%)**	77.7	66
**Non-fermented grains (%)**	4.7	20.5
**Slate grains (%)**	17.6	13.5
**Fermentation index**	1.48	1.29

Table S2. Primers used for 16S rRNA gene and ITS amplicon libraries.

Name	Sequence
515F_L1 (16S rRNA)	ACACTCTTTCCCTACACGACGCTCTTCCGATCTGTGCCAGCMGCCGCGGTAA
806R_L2 (16S rRNA)	GTGACTGGAGTTCAGACGTGTGCTCTTCCGATCTGGACTACHVGGGTWTCTAAT
F_ITS3_KYO2_L1 (ITS)	ACACTCTTTCCCTACACGACGCTCTTCCGATCTGATGAAGAACGYAGYRAA
R_ITS4_KYO3_L2 (ITS)	GTGACTGGAGTTCAGACGTGTGCTCTTCCGATCTCTBTTVCCKCTTCACTCG
PE1-1(Barcode F)	AATGATACGGCGACCACCGAGATCTACACCTTGTCGTACACTCTTTCCCTACACGACGCTCTTCCGATCT
PE1-2 (Barcode F)	AATGATACGGCGACCACCGAGATCTACACACCAGATCACACTCTTTCCCTACACGACGCTCTTCCGATCT
PE1-3 (Barcode F)	AATGATACGGCGACCACCGAGATCTACACTATACCGGACACTCTTTCCCTACACGACGCTCTTCCGATCT
PE1-4 (Barcode F)	AATGATACGGCGACCACCGAGATCTACACCGCGATTAACACTCTTTCCCTACACGACGCTCTTCCGATCT
PE1-5 (Barcode F)	AATGATACGGCGACCACCGAGATCTACACACACGTGTACACTCTTTCCCTACACGACGCTCTTCCGATCT
PE1-6 (Barcode F)	AATGATACGGCGACCACCGAGATCTACACCTGTGTCTACACTCTTTCCCTACACGACGCTCTTCCGATCT
PE1-7 (Barcode F)	AATGATACGGCGACCACCGAGATCTACACGCTTGCTTACACTCTTTCCCTACACGACGCTCTTCCGATCT
PE1-8 (Barcode F)	AATGATACGGCGACCACCGAGATCTACACTCACCTCAACACTCTTTCCCTACACGACGCTCTTCCGATCT
PE1-9 (Barcode F)	AATGATACGGCGACCACCGAGATCTACACGGAATTGGACACTCTTTCCCTACACGACGCTCTTCCGATCT
PE1-10 (Barcode F)	AATGATACGGCGACCACCGAGATCTACACCTAGCTAGACACTCTTTCCCTACACGACGCTCTTCCGATCT
PE1-11 (Barcode F)	AATGATACGGCGACCACCGAGATCTACACTTAAGCGCACACTCTTTCCCTACACGACGCTCTTCCGATCT
PE1-12 (Barcode F)	AATGATACGGCGACCACCGAGATCTACACTGAGCAGAACACTCTTTCCCTACACGACGCTCTTCCGATCT
PE1-13 (Barcode F)	AATGATACGGCGACCACCGAGATCTACACCGGCATTAACACTCTTTCCCTACACGACGCTCTTCCGATCT
PE1-14 (Barcode F)	AATGATACGGCGACCACCGAGATCTACACACCAGTTGACACTCTTTCCCTACACGACGCTCTTCCGATCT
PE1-15 (Barcode F)	AATGATACGGCGACCACCGAGATCTACACCGTTGCTTACACTCTTTCCCTACACGACGCTCTTCCGATCT
PE2-1 (Barcode R)	CAAGCAGAAGACGGCATACGAGATCCTAGCTTGTGACTGGAGTTCAGACGTGTGCTCTTCCGATCT
PE2-2 (Barcode R)	CAAGCAGAAGACGGCATACGAGATTTCCAAGGGTGACTGGAGTTCAGACGTGTGCTCTTCCGATCT
PE2-3 (Barcode R)	CAAGCAGAAGACGGCATACGAGATATCCGCTTGTGACTGGAGTTCAGACGTGTGCTCTTCCGATCT
PE2-4 (Barcode R)	CAAGCAGAAGACGGCATACGAGATCTGTGTGAGTGACTGGAGTTCAGACGTGTGCTCTTCCGATCT
PE2-5 (Barcode R)	CAAGCAGAAGACGGCATACGAGATCTGTCTGTGTGACTGGAGTTCAGACGTGTGCTCTTCCGATCT
PE2-6 (Barcode R)	CAAGCAGAAGACGGCATACGAGATTACGGCTAGTGACTGGAGTTCAGACGTGTGCTCTTCCGATCT
PE2-7 (Barcode R)	CAAGCAGAAGACGGCATACGAGATCGTTGCAAGTGACTGGAGTTCAGACGTGTGCTCTTCCGATCT

PE2-8 (Barcode R)	CAAGCAGAAGACGGCATACGAGATCTGAAGTCGTGACTGGAGTTCAGACGTGTGCTCTTCCGATCT
PE2-9 (Barcode R)	CAAGCAGAAGACGGCATACGAGATTCTCCTCTGTGACTGGAGTTCAGACGTGTGCTCTTCCGATCT
PE2-10 (Barcode R)	CAAGCAGAAGACGGCATACGAGATCTCAAGACGTGACTGGAGTTCAGACGTGTGCTCTTCCGATCT
PE2-11 (Barcode R)	CAAGCAGAAGACGGCATACGAGATTCCTAGCTGTGACTGGAGTTCAGACGTGTGCTCTTCCGATCT
PE2-12 (Barcode R)	CAAGCAGAAGACGGCATACGAGATTCCTCTTCGTGACTGGAGTTCAGACGTGTGCTCTTCCGATCT
PE2-13 (Barcode R)	CAAGCAGAAGACGGCATACGAGATGCATAACCGTGACTGGAGTTCAGACGTGTGCTCTTCCGATCT
PE2-14 (Barcode R)	CAAGCAGAAGACGGCATACGAGATACCTGTAGGTGACTGGAGTTCAGACGTGTGCTCTTCCGATCT
PE2-15 (Barcode R)	CAAGCAGAAGACGGCATACGAGATGTGAGAGTGTGACTGGAGTTCAGACGTGTGCTCTTCCGATCT

Table S3. Composition of GYE 1 and GYE 2 media.

Components	GEY 1 (w/v)	GEY 2 (w/v)
Glucose	10%	5%
Yeast extract	1%	1%
$CaCO_3$	2%	0,5%
Agar	1,5	1,5%

Table S4. Bioinformatics parameters in the workflow for detecting inter-specific microbiome communities.

Software parameter				Molecular marker	
				**16S rRNA gene**	**ITS**
Pre-processing of reads	***Trimmomatic***		Leading and trailig	5	5
			headcrop	27	18
			slidingwindow	4:25	7:18
			minlen	150	150
	QIIME version 1.9.1	*multiple_join_paired_ends*	method	fastq-join	
			min_overlap	100	20
			perc_max_diff	10	5
		multiple_split_libraries_fastq	max_bad_run_length	0	0
			phred_quality_threshold	19	15
			min_per_read_length_fraction	0.90	0.60
OTU detection		*pick_otus*	method	UCLUST	
			similarity	0.97	0.95
			optimal_uclust	active	
		assign_taxonomy	method	UCLUST	BLASTn
		filter_otus_from_otu_table	min_count_fraction	0.1	0.01
		filter_taxa_from_otu_table	negative_taxa	Chloroplast	no used

Table S5. Description of the parameter used for oligotype delineation.

OTU	Oligotype	bp of oligotype	Number of position	Shannon-entropy values cut-off	Montaña Santandereana (MS)																																											
					Dry season																				Wet season																							
					Upper section										Middle section										Upper section												Middle section											
					0	12	24	36	48	60	72	84	96	108	0	12	24	36	48	60	72	84	96	108	0	12	24	36	48	60	72	84	96	108	120	132	0	12	24	36	48	60	72	84	96	108	120	132
Enterobacteriaceae	Enterobacteriaceae-1	CCGAA	5	0.1																																												
	Enterobacteriaceae-2	CCAAA																																														
	Enterobacteriaceae-3	CTGAA																																														
	Enterobacteriaceae-4	TTGAA																																														
	Enterobacteriaceae-5	CCGAG																																														
	Enterobacteriaceae-6	CCAAG																																														
Lactobacillaceae	Lactobacillaceae-1	GCGTCGCTA	9	0.5				0								0												0										0		0								
	Lactobacillaceae-2	GCGTCGCCA																										0																				
	Lactobacillaceae-3	ACGTTGCCA																										0																				
	Lactobacillaceae-4	GCGTTGCTA						0																																								
	Lactobacillaceae-5	GCGTTGCCA						0																																								
	Lactobacillaceae-6	GGA-CGCTA																																														
	Lactobacillaceae-7	GCGTCCTTG																																														
	Lactobacillaceae-8	ACGTCGCTA						0																																								
	Lactobacillaceae-9	ACGTCGCCA																																														
	Lactobacillaceae-10	GCGTCGCTG																																														
	Lactobacillaceae-11	ACGTTCTTG																																														
	Lactobacillaceae-12	GCGTCCCTG																																														
Lactobacillus	Lactobacillus-1	GGA-TCAGACTTG	13	0.2																																												
	Lactobacillus-2	GGA-CTGGACTCG																																														
	Lactobacillus-3	GCGTTCAGACTTG																																														
	Lactobacillus-4	GGA-CTGGACTTG																																														
	Lactobacillus-5	GGA-TCAGGGCTA																																														
	Lactobacillus-6	GGA-TTAGACTCG																																														
	Lactobacillus-7	GGA-TCAGGCCTG																																														
Fructobacillus	Fructobacillus-1	CCGACTG	7	0.1											1																																	
	Fructobacillus-2	CCGATTG																																														
	Fructobacillus-3	GGAGCTG																																														
	Fructobacillus-4	CCGACCA																																														
Acetobacteraceae	Acetobacteraceae-1	AACCGCG	7	0.6																																												
	Acetobacteraceae-2	AACCGCT																																														
	Acetobacteraceae-3	ATTCACG																																														
	Acetobacteraceae-4	ATTCGCG																																														
	Acetobacteraceae-5	ATTCACT																																														
	Acetobacteraceae-6	AACCCAT																																														
	Acetobacteraceae-7	ATATGAT																																														
	Acetobacteraceae-8	ATTCGCT																																														
	Acetobacteraceae-9	TACCGCT																																														
	Acetobacteraceae-10	ATATGCG																																														
	Acetobacteraceae-11	ATTCCAT																																														
	Acetobacteraceae-12	TTTCACT																																														
Acetobacter	Acetobacter-1	TGTGCAT	7	0.1					1	1	1	1	1	1			1	1	1	1	1	1	1	1					1	1	1	1	1	1	1	1					1	1	1	1	1	1	1	1
	Acetobacter-2	TGTGCAG																																														
	Acetobacter-3	TACGTAT																																														
	Acetobacter-4	TACGCAT																																														
	Acetobacter-5	TGTGCAA																																														
	Acetobacter-6	TGTGGCG																																														
	Acetobacter-7	TTGTAAT																																														

OTU	Oligotype	bp of oligotype	Number of position	Shannon-entropy values cut-off
Enterobacteriaceae	Enterobacteriaceae-1	CCGAA	5	0.1
	Enterobacteriaceae-2	CCAAA		
	Enterobacteriaceae-3	CTGAA		
	Enterobacteriaceae-4	TTGAA		
	Enterobacteriaceae-5	CCGAG		
	Enterobacteriaceae-6	CCAAG		
Lactobacillaceae	Lactobacillaceae-1	GCGTCGCTA	9	0.5
	Lactobacillaceae-2	GCGTCGCCA		
	Lactobacillaceae-3	ACGTTGCCA		
	Lactobacillaceae-4	GCGTTGCTA		
	Lactobacillaceae-5	GCGTTGCCA		
	Lactobacillaceae-6	GGA-CGCTA		
	Lactobacillaceae-7	GCGTCCTTG		
	Lactobacillaceae-8	ACGTCGCTA		
	Lactobacillaceae-9	ACGTCGCCA		
	Lactobacillaceae-10	GCGTCGCTG		
	Lactobacillaceae-11	ACGTTCTTG		
	Lactobacillaceae-12	GCGTCCCTG		
Lactobacillus	Lactobacillus-1	GGA-TCAGACTTG	13	0.2
	Lactobacillus-2	GGA-CTGGACTCG		
	Lactobacillus-3	GCGTTCAGACTTG		
	Lactobacillus-4	GGA-CTGGACTTG		
	Lactobacillus-5	GGA-TCAGGGCTA		
	Lactobacillus-6	GGA-TTAGACTCG		
	Lactobacillus-7	GGA-TCAGGCCTG		
Fructobacillus	Fructobacillus-1	CCGACTG	7	0.1
	Fructobacillus-2	CCGATTG		
	Fructobacillus-3	GGAGCTG		
	Fructobacillus-4	CCGACCA		
Acetobacteraceae	Acetobacteraceae-1	AACCGCG	7	0.6
	Acetobacteraceae-2	AACCGCT		
	Acetobacteraceae-3	ATTCACG		
	Acetobacteraceae-4	ATTCGCG		
	Acetobacteraceae-5	ATTCACT		
	Acetobacteraceae-6	AACCCAT		
	Acetobacteraceae-7	ATATGAT		
	Acetobacteraceae-8	ATTCGCT		
	Acetobacteraceae-9	TACCGCT		
	Acetobacteraceae-10	ATATGCG		
	Acetobacteraceae-11	ATTCCAT		
	Acetobacteraceae-12	TTTCACT		
Acetobacter	Acetobacter-1	TGTGCAT	7	0.1
	Acetobacter-2	TGTGCAG		
	Acetobacter-3	TACGTAT		
	Acetobacter-4	TACGCAT		
	Acetobacter-5	TGTGCAA		
	Acetobacter-6	TGTGGCG		
	Acetobacter-7	TTGTAAT		

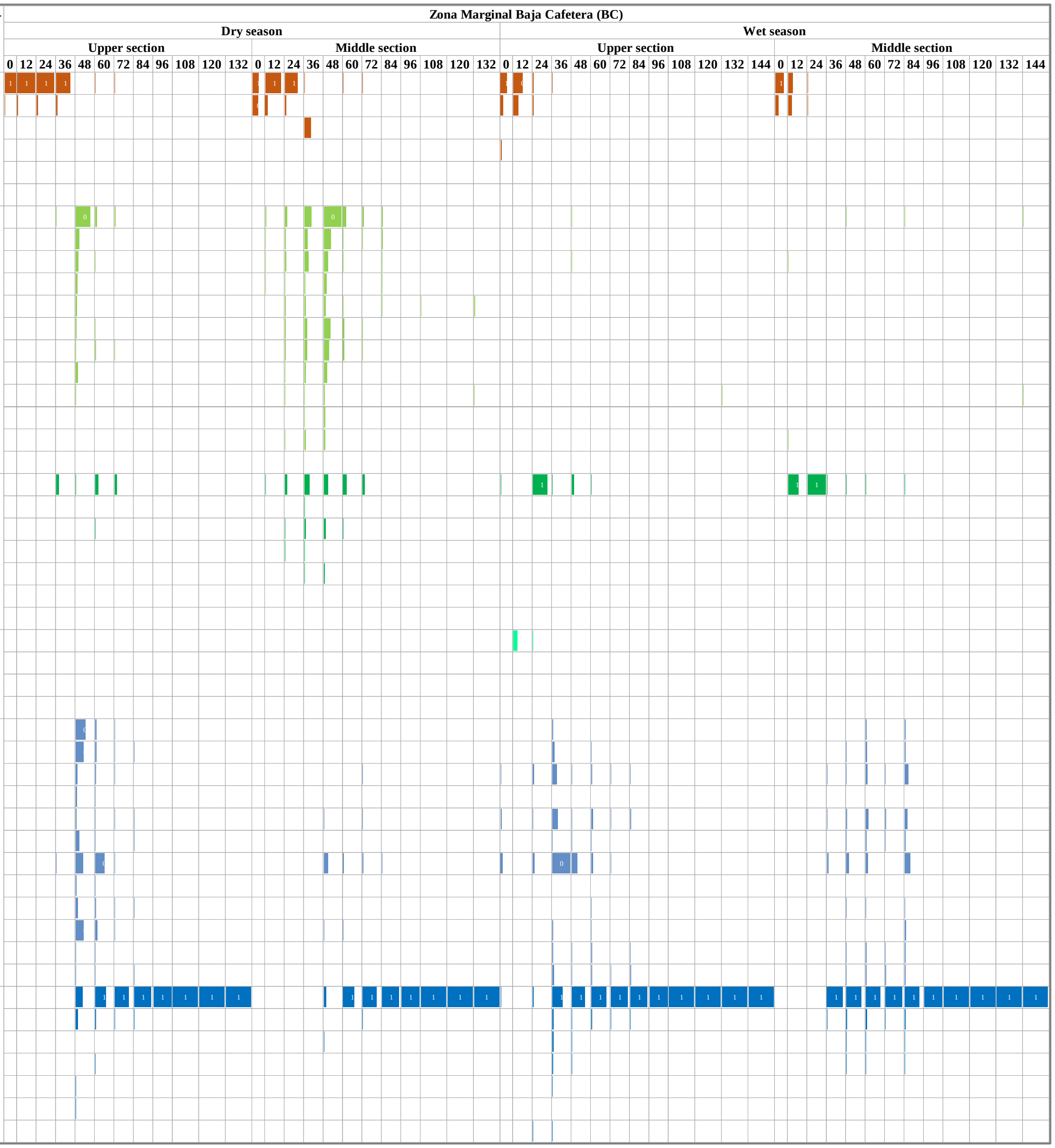

The nucleotides identified as delimiter of each oligotype are shown (pb of oligotype), as well as the numbers of position and the cut-off values for the Shannon entropy. The horizontal bars at the right part of the table represents the relative abundance of oligotypes at each time point.

Table S6. Relationship between oligotypes, isolates and taxonomic affiliation.

Oligotype	Bacterial isolates identifier*	Closest related taxonomic group**
**Enterobacteracea-1**	300	*Pantoea gavinae, Enterobacter hormaechei*
**Enterobacteracea-2**	**NO**	*Tatumella ptyseos*
**Enterobacteracea-3**	299	*Enterobacter cloacae*
**Enterobacteracea-4**	**NO**	*Tatumella terrea*
**Enterobacteracea-5**	300	*Pantoea gavinae, Enterobacter hormaechei*
**Enterobacteracea-6**	**NO**	*Tatumella terrea*
**Acetobacter-1**	28, 35, 36, 40, 41, 43, 45, 57, 61, 311	*Acetobacter pasteurianus*
**Acetobacter-2**	307, 310, 312, 313	*Acetobacter tropicales, A. senegalensis*
**Acetobacter-3**	**NO**	**NC**
**Acetobacter-4**	**NO**	**NC**
**Acetobacter-5**	**NO**	**NC**
**Acetobacter-6**	**NO**	**NC**
**Acetobacter-7**	**NO**	**NC**
**Acetobacteraceae-1**	**NO**	*Komagataeibacter oboediens*
**Acetobacteraceae-2**	**NO**	*Komagataeibacter oboediens*
**Acetobacteraceae-3**	**NO**	*Ameyamaea chiangmaiensis*
**Acetobacteraceae-4**	**NO**	*Gloconobacter entanii*
**Acetobacteraceae-5**	**NO**	*Ameyamaea chiangmaiensis*
**Acetobacteraceae-6**	**NO**	**NC**
**Acetobacteraceae-7**	**NO**	**NC**
**Acetobacteraceae-8**	**NO**	*Gloconobacter entanii*
**Acetobacteraceae-9**	**NO**	*Komagataeibacter oboediens*
**Acetobacteraceae-10**	**NO**	*Komagataeibacter europaeus*
**Acetobacteraceae-11**	**NO**	**NC**
**Acetobacteraceae-12**	**NO**	*Ameyamaea chiangmaiensis*
**Fructobacillus -1**	**NO**	*Fructobacillus pseudoficulneus*
**Fructobacillus -2**	**NO**	*Fructobacillus pseudoficulneus*
**Fructobacillus -3**	34	*Leuconostoc citreum*
**Fructobacillus -4**	**NO**	*Fructobacillus pseudoficulneus*
**Lactobacillus-1**	**NO**	*Lactobacillus cacaonum*
**Lactobacillus-2**	19	*Lactobacillus nagelii*
**Lactobacillus-3**	**NO**	**NC**
**Lactobacillus-4**	19	*Lactobacillus nagelii*

**Lactobacillus-5**	24, 46	*Lactobacillus previs, Lactobacillus farraginis*
**Lactobacillus-6**	49	*Lactobacillus ghanensis*
**Lactobacillus-7**	**NO**	*Lactobacillus cacaonum*
**Lactobacillaceae-1**	2, 18, 56, 308,309,314,315	*Lactobacillus plantarum, Lactobacillus fermentum*
**Lactobacillaceae-2**	**NO**	**NC**
**Lactobacillaceae-3**	209	*Lactobacillus rhamnosus, Lactobacillus cameliae*
**Lactobacillaceae-4**	**NO**	*Lactobacillus plantarum*
**Lactobacillaceae-5**	**NO**	*Uncultured Lactobacillus present in Drosophila suzukii*
**Lactobacillaceae-6**	19, 308	*Lactobacillus plantarum, Lactobacillus fermentum*
**Lactobacillaceae-7**	**NO**	*Lactobacillus plantarum*
**Lactobacillaceae-8**	**NO**	**NC**
**Lactobacillaceae-9**	6, 13, 37, 44, 227	*Pediococcus acidilactici, Pediococcus pentosaceus*
**Lactobacillaceae-10**	19, 308	*Lactobacillus plantarum, Lactobacillus fermentum*
**Lactobacillaceae-11**	**NO**	*Lactobacillus plantarum*
**Lactobacillaceae-12**	**NO**	*Lactobacillus plantarum*

***** The isolates where designated to oligotypes based on the evolutionary relationship obtained in the phylogenetic tree whenever the phylogenetic distant between oligotypes and isolates was less than 1%. ****** The closest related taxonomic groups where obtained using NCBI nr best hit with less than 1% sequence differences. **NO** (Not obtained). **NC** (Not-closely related sequences in the databases).
